# *kretschmanni*’s global relatives: The *Aphanogmus* species with spiny sternite females (Hymenoptera, Ceraphronidae)

**DOI:** 10.3897/zookeys.1286.193282

**Published:** 2026-07-27

**Authors:** Jonah A. Schneider, Tobias Salden, Marina Moser, Ralph S. Peters

**Affiliations:** 1 Leibniz Institute for the Analysis of Biodiversity Change, Museum Koenig Bonn, Adenauerallee 160, 53113 Bonn, Germany Leibniz Institute for the Analysis of Biodiversity Change, Museum Koenig Bonn Bonn Germany; 2 State Museum of Natural History Stuttgart, Rosenstein 1, 70191 Stuttgart, Germany State Museum of Natural History Stuttgart Stuttgart Germany

**Keywords:** CO1 barcodes, Integrative taxonomy, Morphology, new species, parasitoid wasps, Waterston's evaporatorium

## Abstract

The Western Palaearctic *Aphanogmus kretschmanni* Moser, 2023 is unique among all described species of Ceraphronidae in having two distinct rows of spines on the seventh metasomal sternite of the female. In this study, we examined additional *Aphanogmus* that share this trait and found at least five additional putative species in an integrative taxonomy approach using morphology and publicly available *CO1* barcode data on BOLD. Two new species are described herein: *A. manta* Schneider, Salden & Peters, **sp. nov**. and *A. liceodosriosi* Schneider, Salden & Peters, **sp. nov**. The diagnosis of *A. kretschmanni* is updated. The remaining putative species are listed but not formally described as reliable diagnoses require additional material. We also describe males, including male genitalia, of species with spiny sternite females for the first time. Males lack the metasomal sternal spines which implies a sex-specific function of the spines, for example, in oviposition. In our morphological diagnoses, we use characters of the Waterston’s evaporatorium (WE) present on the sixth metasomal tergite in both males and females in addition to previously established morphological characters and further highlight the value of the WE in delimiting Ceraphronidae species and morphologically matching males and females. The number of putative species and their distribution literally around the globe (Neotropics, Afrotropics, Palaearctic, Australasia, Orientalis) imply the presence of an unexpectedly large number of relatives of *A. kretschmanni*. Future investigations are necessary to capture the true diversity within the genus *Aphanogmus* and to unravel the biological and evolutionary background of the intriguing sternal spines.

## Introduction

Ceraphronidae are one of the most understudied families of parasitoid wasps. It contains around 430 described species in 16 genera (Johnson and Musetti 2004; Mikó and Deans 2009; Mikó et al. 2018; Salden and Peters 2023). After *Ceraphron*, *Aphanogmus* Thomson, 1858 is the most species-rich genus within the Ceraphronidae, and its species are distributed worldwide (Johnson and Musetti 2004). Approximately 140 species of *Aphanogmus* have been described, with 67 occurring in the Palaearctic realm, 47 in the Afrotropical realm, 16 in the Nearctic realm, 15 in the Oriental realm, seven in the Australasian and Oceanian realm, and three in the Neotropical realm (Johnson and Musetti 2004; Evans et al. 2005; Buhl et al. 2010; Matsuo et al. 2016; Moser et al. 2023; Ranjith et al. 2023; Salden and Peters 2023; Mollaei et al. 2025; Salden et al. 2026a). Nogueira (2008) mentioned 23 additional species from the Neotropical realm that were not formally named.

In 2023 a new species of *Aphanogmus* with a conspicuous trait was described from southern Germany: *Aphanogmus kretschmanni* Moser, 2023 (Moser et al. 2023). So far, only female specimens of the species are known, which bear two prominent ventral rows of spines on the seventh metasomal sternite. This character is not known from any other ceraphronid or Hymenoptera species. We searched the BOLD database image browser (https://www.boldsystems.org; Ratnasingham et al. 2024) for additional specimens of *Aphanogmus* showing this trait. All retrieved specimens, as well as all specimens assigned to the same BINs (including males), were analysed using an integrative taxonomic approach based on morphology and available DNA barcodes.

Morphologically, species of Ceraphronidae are delimited and diagnosed mostly based on the male genitalia, a character that is not based on size-dependent characters like body shape or sculpture that can be affected by allometry (Mikó et al. 2013; Salden and Peters 2023). Females exhibit comparatively few diagnostic characters and are therefore often difficult to diagnose morphologically, although many species were historically described based on females (Johnson and Musetti 2004). Ulmer et al. (2021) proposed the Waterston’s evaporatorium (WE) as an additional diagnostic morphological structure present in both male and female Ceraphronidae. The Waterston’s evaporatorium is a synapomorphic trait of the Ceraphronidae and is located medially on the sixth metasomal tergite (Mikо and Deans 2009; Ulmer et al. 2021). Waterston (1923) presumed the evaporatorium to be related to respiration, more recent studies suggest that the Waterston’s evaporatorium is associated with glands and offers an increased evaporative surface for glandular products (Ogloblin 1944; Dessart 1992; Mikó and Deans 2009). Here, we add the Waterston’s evaporatorium to our species delimitation and diagnoses toolkit and further demonstrate its usefulness in Ceraphronidae integrative taxonomy.

The DNA barcode data and morphological examination, including male genitalia and WE, allows for species delimitation and the description of two new species. Additionally, we assess the global diversity of *Aphanogmus* species with metasomal spines in females and contribute to our understanding of the role of these unique spines.

## Material and methods

### Handling of specimens and imaging

The BOLD portal (accessed February 2025) was queried for *Aphanogmus* and all available photos of BINs were checked for specimens with spines on metasomal sternites. All specimens from the following nine BINs which included at least one specimen with a spiny sternite based on available photos were loaned from the Centre for Biodiversity Genomics (CBG):

BOLD:ACP1659, BOLD:ACP4134, BOLD:ACY0736, BOLD:ADJ0332, BOLD:ADU4363, BOLD:AFB8353, BOLD:AFF8142, BOLD:AFS2572, BOLD:AFS2573.

A total of 47 specimens were included in these BINs. Nine specimens could not be located and were neither examined morphologically nor added in the molecular species delimitation. These specimens and their BOLD sample IDs are listed in the material sections of the respective species treatments.

The additional BIN BOLD:AFA2054 contained only one specimen that was not found and examined (BIOUG93948-F12), so this BIN and putative species from Tanzania is not included here.

A total of 38 BOLD specimens from nine BINs were considered for the following investigations. In addition, nine sequences of *Aphanogmus kretschmanni* were added to the DNA barcode dataset. Sequences are also available at BOLD (see Suppl. material 1 for details).

The specimens were collected with Malaise traps in different countries and on different continents.

Additionally, two paratypes (SMNS_Hym_Hym_027509 and SMNS_Hym_Cer_000647) and nine additional specimens of *A. kretschmanni* were morphologically examined. Except for SMNS_Hym_Cer_000647, they all were provided by the State Museum of Natural History in Stuttgart, Germany and include the following IDs:

SMNS_Hym_Cer_000440, SMNS_Hym_Cer_000445, SMNS_Hym_Cer_000446, SMNS_Hym_Cer_000467, SMNS_Hym_Cer_000468, SMNS_Hym_Hym_027357, SMNS_Hym_Hym_027685, SMNS_HYM_080362, SMNS_HYM_080363.

For the metadata of the specimens included, please see Suppl. material 1.

All specimens were transferred from ethanol into glycerol droplets (100%) on concave microscope slides. They were examined and later dissected under a LEICA M205 C stereomicroscope with a LEICA PLANAPO 1.0× ocular and Nikon CFI 10×/22 Microscope Eyepieces. Genitalia of the male specimens were dissected using Dumont Style 5 Inox 02 forceps and a minuten pin (100 µm diameter). The metasomas posterior to the synterga were separated from the rest of the body in all specimens and the Waterston’s evaporatorium bearing tergites were dissected using two minuten pins (100 µm diameter). Then, the dissected tergites were transferred into small glycerol droplets (100%) on a microscope slide. To minimise the risk of damage, the cover slide was placed in the following way: First, one slide each was placed left and right of the droplet and a third one on top of these two. The lateral slides were subsequently removed one by one to allow the top one to smoothly cover the droplet. For long-term storage, Waterston’s evaporatoria were transferred from the glycerol droplets to ethanol and then slide-mounted in Euparal. Photos of specimens of all putative species, as well as of the male genitalia and of the Waterston’s evaporatoria, were taken in glycerol droplets (if not stated otherwise) using a Keyence VHX-7100 microscope. All measurements of body, male genitalia, and Waterston’s evaporatoria were done using the same device and its implemented software. The images were edited using GNU image manipulation Program v. 3.0.4 (GIMP, available at https://www.gimp.org/) and assembled into image plates using Inkscape v. 1.4 (available at https://inkscape.org).

### Morphological characters and species descriptions

The species descriptions are based on more than one specimen if available, indicated by a number in parentheses (*n* = X). Characters of the holotype are given in parentheses in the respective character description. Terminology follows the Hymenoptera Anatomy Ontology (HAO) (Yoder et al. 2010, available online at http://portal.hymao.org, accessed in August 2025), Salden and Peters (2023, 2025), and Salden et al. (2026b). The anatomical structures of the Waterston’s evaporatorium are listed, defined, and referenced in Table 1.

**Table 1.** List of anatomical structures of the Waterston’s evaporatorium that are used in the species descriptions.

**Character**	**Definition**
acrotergal calyx	The concave area on the acrotergite on metasomal tergite 6 that bears the orifice of type III exocrine glands (modified, from HAO portal).
bulla of the Waterston‘s evaporatorium	The smooth, median area on the anterior part of the Waterston‘s evaporatorium posterior to the antecosta (from HAO portal).
campaniform sensillum	The aporous sensillum with a flat or dome-shaped, circular cuticular component that is delimited by a shallow depression (from HAO portal).
caudal setal row	Setal row that arises from the posterior ridge of the tergite (from Ulmer et al. 2021).
evaporatorium	The area that is highly sculptured and surrounds the opening of a gland (from HAO portal).
proximomedian lamella of the Waterston’s evaporatorium	The flattened conjunctival invagination at the posterior margin of the Waterston‘s evaporatorium that folds over the posterior region of the evaporatorium (from HAO portal).
tergal apodeme	The projection on the anterior margin of the abdominal tergite that serves as the site of insertion of lateral intertergal retractor muscle and for the intertergal extensor muscle (from HAO portal).
Waterston’s evaporatorium	The evaporatorium that is located medially on the acrotergite of metasomal tergite 6 (modified, from HAO portal).
WE length	The median anatomical line between the anteriormost point of the acrotergal calyx and the posteriormost point of the evaporatorium (Salden et al. 2026b).
WE length/median T6 length	The WE length divided by the length of the metasomal tergite 6, measured as the median anatomical line between the anteriormost point of the acrotergal calyx and the posterior margin of the tergite (Salden et al. 2026b).

### DNA sequencing and molecular species delimitation

The DNA barcode sequences of the specimens were generated by the Canadian Centre for DNA Barcoding (CCDB). The DNA extraction was performed following the protocol by Ivanova et al. (2006). Subsequently, the DNA barcode sequences were amplified following the “CCDB CO1 Amplification Protocol” with a LepF1 forward and a LepR1 reverse primer (Hebert et al. 2004). Sequencing followed the “CCDB Sequencing Protocol” and the sequences were then cleaned following the clean-up Protocol by Grainger and Ivanova (2007). All CCDB Protocols are available online at https://ccdb.ca/resources/ (accessed on 26 August 2025).

The sequences of *A. kretschmanni* were taken from the BOLD dataset provided by Moser et al. (2023; https://doi.org/10.5883/DS-CERAPKR). One of the sequences of *A. kretschmanni* was removed from the alignment due to its short sequence length (SMNS_Hym_Cer_00465, BOLD Sample ID: SMNS_1179428). The SMNS_Hym-IDs (used here) can be assigned to the corresponding BOLD Sample IDs using the publication by Moser et al. (2023). Two additional sequences of *A. kretschmanni* from BOLD were added (accessed May 2025, online available at: https://portal.boldsystems.org/record/SMNSC1217-25; https://portal.boldsystems.org/record/SMNSC1203-25). The barcode of the outgroup (NOCER-637 *Lagynodes acuticornis*) was also downloaded from the BOLD portal (https://portal.boldsystems.org/record/NOMEG637-22#COI-5P).

The sequences were aligned with MUSCLE (Edgar 2004) in AliView v. 1.28 (Larsson 2014). Species delimitation was performed using the ASAP algorithm (default settings, using the first ranked result, Puillandre et al. 2021) and objective clustering with SpeciesIdentifier (spID) v. 1.6.2 (Meier et al. 2006) applying a 3% threshold. In addition, a maximum likelihood tree was reconstructed using IQ tree v. 3.0.1 (Wong et al. 2025) and branch support was assessed with 1000 ultrafast bootstrap replicates (Hoang et al. 2018). The tree was rooted in Figtree v. 1.4.4 (Rambaut 2018, available at http://tree.bio.ed.ac.uk/software/figtree/) using the outgroup sequence (NOCER-637 *Lagynodes acuticornis*) obtained from BOLD. The tree was applied to the multi-rate Poisson Tree Processes (mPTP) species delimitation algorithm (Kapli et al. 2017) via the web server (https://mptp.h-its.org/#/tree, default settings, accessed 2^nd^ August 2025). The results of the species delimitation analyses were integrated into the maximum likelihood tree using Inkscape v. 1.4 (Inkscape project).

Consensus sequences were generated for the two newly described species based only on the type material with JalView v. 2.11.4.1 (Waterhouse et al. 2009). A distance matrix was generated with GENEIOUS Prime v. 2022.1.1 (Biomatters Ltd.), from which maximum intraspecific and minimum interspecific distances were extracted, again using only the type material of the newly described species. The number of sequences and the respective closest species are given in parentheses.

### Institutional abbreviations

**BIOUG** Centre for Biodiversity Genomics, University of Guelph, Guelph, Canada;

**CNCI** Canadian National Collection of Insects, Arachnids and Nematodes, Ottawa, Canada;

**KNP** Skukuza Museum, South African National Parks, Kruger National Park, South Africa;

**SMNS** State Museum of Natural History Stuttgart, Stuttgart, Germany;

**ZFMK** Leibniz Institute for the Analysis of Biodiversity Change, Museum Koenig, Bonn, Germany.

## Results

### Molecular analyses

**Figure 1.**
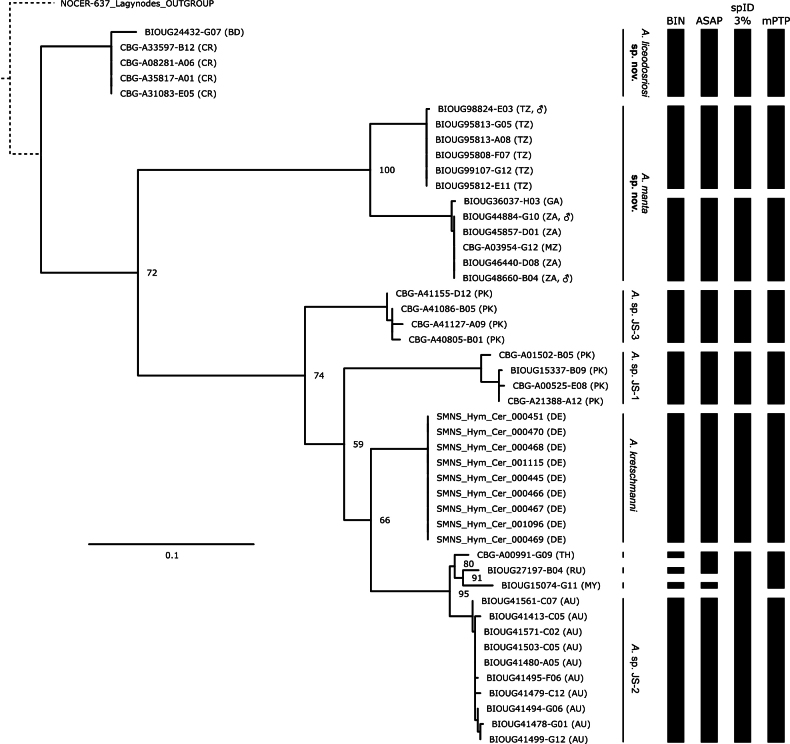
Results from species delimitation using DNA barcode data. Included are female *Aphanogmus* specimens with spines on the 7^th^ metasomal sternite and males assigned to the same BINs on BOLD. Maximum likelihood tree generated with IQ tree, numbers on nodes are ultrafast-bootstrap support values. The results of the four different species delimitation algorithms are shown on the right. The species are named following the results stated below (see taxonomy part). The outgroup (NOCER-637 *Lagynodes acuticornis*) is connected to the remaining tree by a dotted line that is not to scale. The countries from which the specimens originate are indicated in parentheses. In addition, sex is indicated for males, all specimens without indication are female. Country codes: AU=Australia, BD=Bangladesh, CR=Costa Rica, DE=Germany, GA=Gabon, MY=Malaysia, MZ=Mozambique, PK=Pakistan, RU=Russia, TH=Thailand, TZ=Tanzania, ZA=South Africa.

The molecular analyses are based on an alignment of 48 specimens (47 *Aphanogmus* plus one outgroup; for the alignment including consensus sequences of newly described species see Suppl. material 2). The length of the sequences varied between 447 bp and 661 bp. BOLD suggests ten different BINs including *A. kretschmanni*. Nine different species are suggested by ASAP (first rank). Analyses with mPTP suggest eight species, analyses with SpID (with a 97% threshold) suggest seven species. The difference in number of species between results with the different delimitation tools is based only on the different assignments of three specimens (BIOUG15074-G11, CBG-A00991-G09, and BIOUG27197-B04). Each of the specimens forms a separate BIN (BOLD:ACP1659 with BIOUG15074-G11, BOLD:AFS2572 with CBG-A00991-G09, BOLD:AFS2573 with BIOUG27197-B04). ASAP infers BIOUG15074-G11 as a separate species and lumps the other two, while mPTP lumps all three as one species. SpID combines all three with 10 other specimens which are inferred as a separate species by all other methods (Fig. 1).

### Taxonomy

#### Aphanogmus manta

Taxon classificationAnimaliaHymenopteraCeraphronidae

Schneider, Salden & Peters sp. nov.

5D50D80A-805B-5100-80C4-492037A74633

https://zoobank.org/40968D7D-2D10-41CE-867D-CF7CFDB94AD6

Figs 2, 3

##### Type material.

***Holotype*** South Africa • 1 ♂; Mpumalanga, Kruger National Park, Marula North, Satara; 24.397°S, 31.777°E; 264 m a.s.l.; 20. May 2019; Tibane A. leg.; Malaise trap; BOLD Sample ID: BIOUG48660-B04; ZFMKZFMK-Hym-00043047. ***Paratypes*** South Africa • 1 ♂; Mpumalanga, Kruger National Park, Marula North, Nwanetsi; 24.472°S, 31.978°E; 173 m a.s.l.; 23. Jul. 2018; Bryden R. leg.; Malaise trap; BOLD Sample ID: BIOUG44884-G10; KNP • 1 ♀; Mpumalanga, Kruger National Park, Marula South, Lower Sabie; 25.123°S, 31.916°E; 178 m a.s.l.; 21. Sep. 2018; Mathoho C. leg.; Malaise trap; BOLD Sample ID: BIOUG45857-D01; CNCI • 1 ♀; Limpopo, Kruger National Park, Nxanatseni North, Pafuri; 22.42°S, 31.2294°E; 158 m a.s.l.; 13. Nov. 2018; Baloyi G. leg.; Malaise trap; BOLD Sample ID: BIOUG46440-D08; CNCI. Gabon • 1 ♀; Moyen-Ogooue, Lope National Park, Rocher du Miel, Savannah; 0.165°S, 11.617°E; 240 m a.s.l.; 26. Dec. 2014; Decaens T. leg.; Malaise trap; BOLD Sample ID: BIOUG36037-H03; ZFMKZFMK-Hym-00043048. Mozambique • 1 ♀; Sofala, Gorongosa National Park, 2 km W of Chitengo Camp; 18.98°S, 34.33°E; 34 m a.s.l.; 6. Sep. 2019; Hutchinson M., Pringle R. leg.; Malaise trap; BOLD Sample ID: CBG-A03954-G12; ZFMKZFMK-Hym-00043051.

**Figure 2.**
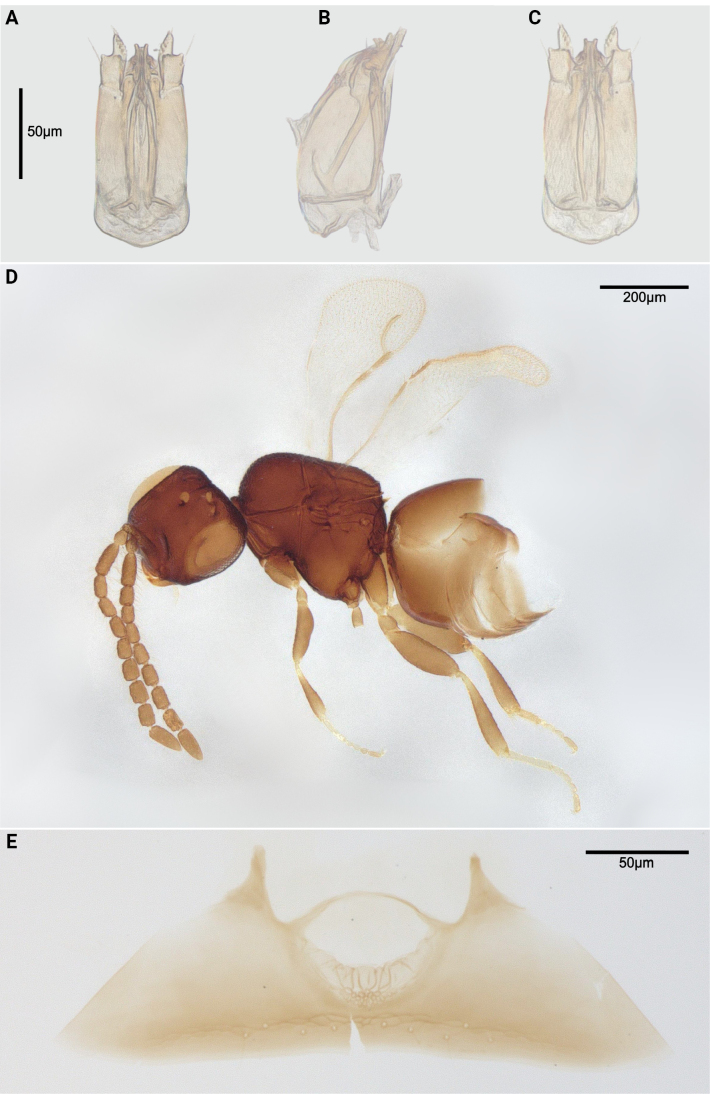
Male *Aphanogmus manta* sp. nov. (holotype: BIOUG48660-B04). **A–C**. Male genitalia in ventral (**A**), lateral (**B**), and dorsal (**C**) view; **D**. Habitus in lateral view; **E**. Waterston’s evaporatorium in dorsal view.

**Figure 3.**
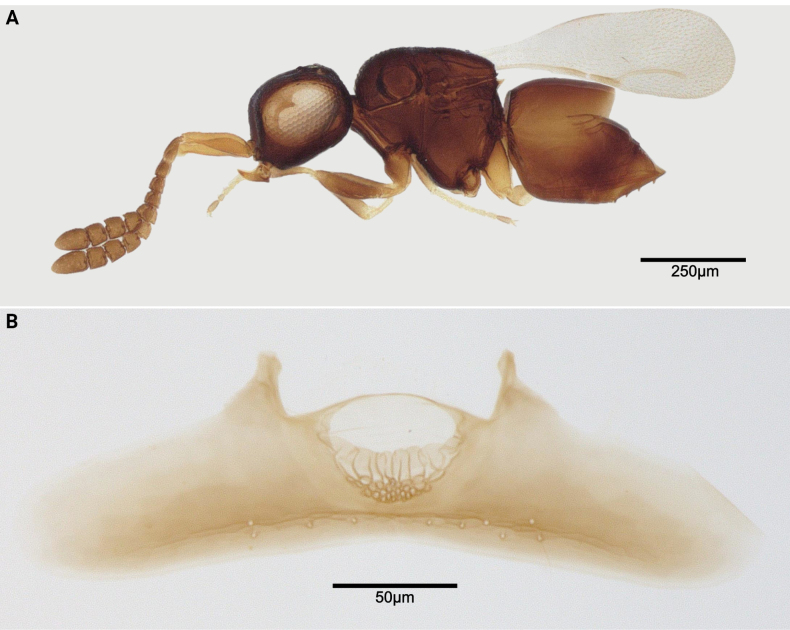
Female *Aphanogmus manta* sp. nov. (paratype: CBG-A03954-G12). **A**. Habitus in lateral view; **B**. Waterston’s evaporatorium in dorsal view.

##### Additional material.

Tanzania • 1 ♂; Mara, Serengeti National Park, Ikoma Gate; 2.189°S, 34.724°E; 1352 m a.s.l.; 24. Jun. 2020; Masoy J. H. leg.; Malaise trap; BOLD Sample ID: BIOUG98824-E03; BIOUG • 1 ♀; Mara, Serengeti National Park, Mbuzi Mawe tented camp; 2.224°S, 34.968°E; 1578 m a.s.l.; 19. Nov. 2021; Masoy J. H. leg.; Malaise trap; BOLD Sample ID: BIOUG99107-G12; BIOUG • 4 ♀♀; Mara, Serengeti National Park, SQ7 Seronera; 2.438°S, 34.849°E; 1525 m a.s.l.; 23 Aug. 2020; Masoy J. H. leg.; Malaise trap; BOLD Sample IDs: BIOUG95813-G05, BIOUG95813-A08, BIOUG95812-E11, BIOUG95808-F07; BIOUG.

##### Note.

There are three additional sequences available on BOLD assigned to BIN BOLD:AFB8353 that could not be located and not examined morphologically and that were not included in the DNA barcode analyses but may represent additional records of *A. manta* sp. nov. BOLD Sample IDs: BIOUG97138-C05, BIOUG95813-E03, BIOUG97138-G06.

##### Diagnosis.

Male with genitalia bilobed, with a trapezoidal ventral lobe and a very short dorsolateral lobe in ventral view as well as additional characters discussed in the diagnostic comparison with similar species section below (Fig. 2A, C). Female with five to nine spines arranged in two rows on the ventral edge of the seventh metasomal sternite (Fig. 3A). Both sexes with mesoscutellum minimally projecting posteriorly compared to the metanoto-propodeo-metapecto-mesopectal complex in lateral view (Fig. 9B), mesometapleuron with indistinct longitudinal striations in its dorsal half, and the Waterston’s evaporatorium (WE) with WE length/median T6 length >0.66, the combined shape of the bulla and the evaporatorium bulbous, one pair of campaniform sensillae present, virtually parallel tergal apodemes and a very distinct proximomedial lamella anteriorly to the evaporatorium (Figs 2E, 3B).

##### Description.

**Male (*n* = 2). *Colouration***. Head, mesosoma and metasoma brown; scape, pedicel, and flagellum pale brown; legs pale brown; wings hyaline; wing venation pale brown; stigmal vein pale brown. ***Body length***. 0.85–0.95 mm (holotype: 0.95 mm). ***Antennae***. Flagellomeres trapezoidal, F1 longer than all other flagellomeres, except F9 (equal in length). ***Mesosoma***. Median mesoscutal line complete. Scutoscutellar sulcus medially acutely angled, scutoscutellar sulcus and transscutal articulation not adjacent, interaxillar sulcus present. Circumscutellar carina present on mesoscutellum, ventral margin of mesoscutellum with distinct angular carina; mesoscutellum minimally projecting posteriorly compared to the metanoto-propodeo-metapecto-mesopectal complex in lateral view; anteromedian projection of the metanoto-propodeo-metapecto-mesopectal complex straight and tapered in lateral view, mesometapleural sulcus present in dorsal half; mesometapleuron with indistinct longitudinal striations in dorsal half; metanotal-propodeal sulcus foveate (Fig. 2D). ***Metasoma***. Basal transverse carina on syntergum present; spines on ventral edge of seventh metasomal sternite absent. ***Waterston’s evaporatorium***. Acrotergal calyx distinctly convex; evaporatorium distinctly convex posteriorly, containing 16 to 24 cells (24); with very distinct proximomedial lamella, extending far beyond the evaporatorium anteriorly; cell size equal to setal socket size; the combined shape of the bulla and the evaporatorium cells is bulbous; bulla large, convex anteriorly and posteriorly, laterally tapered (lemon-shaped); WE length/median T6 length 0.67–0.72 (0.72); tergal apodemes virtually parallel, generally broad; six or seven setae in caudal setal row (7); one pair of campaniform sensillae present (Fig. 2E). ***Male genitalia***. Gvc length between one-fifth and one-quarter of mesonotum length; gvc width 0.68–0.72 × (0.72) gvc length; harpe/gvc index 0.49–0.52 (0.49); harpe bilobed and slightly oriented distoventrally; ventral lobe trapezoidal and dorsolateral lobe very short in ventral view; apex of ventral lobe pointed and of dorsolateral lobe slightly pointed; lateral margin of harpe slightly convex in basal half and straight in apical half, with distinct rectangular emargination (sometimes with visible delineation, see holotype) at mid-length of lateral margin; ventromedial margin of ventral lobe diverging distolaterally (Fig. 2A, C); ventral margin of harpe virtually straight and dorsal margin slightly concave in lateral view (Fig. 2B); ventral lobe length/harpe length 0.37; width of harpe (at rectangular emargination) 0.25–0.32 × (0.25) gvc width and 0.37–0.42 × (0.37) harpe length; harpe with short median setae on ventral lobe, oriented distomedially (with indistinct number), one apical seta on dorsolateral lobe around two-thirds as long as harpe length and one lateral seta of equal length at mid-length of lateral margin of harpe (Fig. 2A, C); aedeagus + gonossiculus length 0.47–0.61 × (0.61) harpe length; gonossiculus with two dorsally oriented digital teeth, apical digital tooth broader and dorsally protruding beyond harpe, basal digital tooth narrower and longer, not dorsally protruding beyond harpe, oriented proximodorsally (Fig. 2B).

**Female (*n* = 4). *Colouration***. Head dark brown; mesosoma dark brown to brown; metasoma brown to pale brown; scape pale brown-yellowish; pedicel pale brown-yellowish; flagellum pale brown; legs brown, to pale brown-yellowish; wings hyaline; wing venation pale brown; stigmal vein pale brown. ***Body length***. 0.80–1.25 mm. ***Mesosoma***. Median mesoscutal line complete. Scutoscutellar sulcus medially acutely angled, scutoscutellar sulcus and transscutal articulation not adjacent, interaxillar sulcus present. Circumscutellar carina present on mesoscutellum, ventral margin of mesoscutellum with distinct angular carina; mesoscutellum minimally projecting posteriorly compared to the metanoto-propodeo-metapecto-mesopectal complex in lateral view; anteromedian projection of the metanoto-propodeo-metapecto-mesopectal complex straight and tapered in lateral view, mesometapleural sulcus present in dorsal half (Fig. 3A); mesometapleuron with indistinct longitudinal striations in dorsal half (Fig. 8B); metanotal-propodeal sulcus foveate (Fig. 3A). ***Metasoma***. Basal transverse carina on syntergum present; ventral edge of seventh metasomal sternite with five to nine spines in two rows, anteriormost two spines situated directly next to each other (Fig. 3A). ***Waterston’s evaporatorium***. Acrotergal calyx convex; evaporatorium distinctly convex posteriorly, containing 17 to 60 cells; with very distinct proximomedial lamella, extending far beyond the evaporatorium anteriorly; cell size equal to setal socket size; the combined shape of the bulla and the evaporatorium cells is bulbous; bulla large, convex posteriorly and anteriorly, laterally slightly tapered; WE length/median T6 length 0.70–0.77; tergal apodemes virtually parallel; 6 to 13 setae in caudal setal row; one pair of campaniform sensillae present (Fig. 3B).

##### *CO1* barcode.

Maximum intraspecific distance: 1.41% (*n* = 6). Minimum interspecific distance (to *Aphanogmus liceodosriosi* sp. nov.): 14.77%. This species corresponds to BIN BOLD:ADJ0332 and BIN BOLD:AFB8353 (see Remarks below).

##### Etymology.

The name refers to the shape of the Waterston’s evaporatorium and the sixth metasomal tergite that is reminiscent of a manta ray (Chondrichthyes: Myliobatiformes: *Mobula* spp.).

##### Distribution.

Afrotropical: Gabon, Mozambique, South Africa (Other Material from Tanzania, see Remarks).

##### Ecology.

Unknown.

##### Remarks.

**Figure 4.**
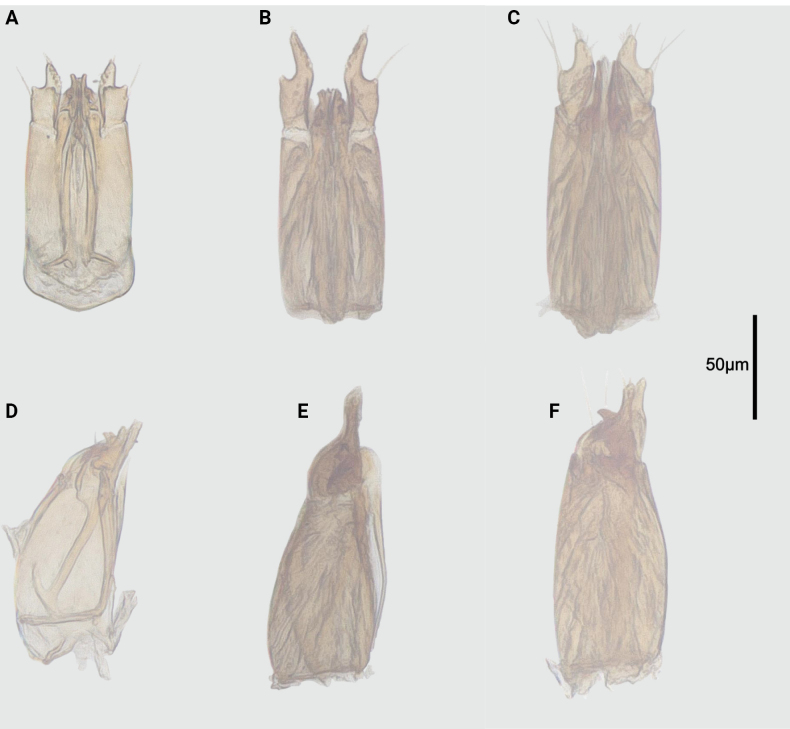
Comparison of the male genitalia of *A. manta* sp. nov. (**A, D**) with those of similar Afrotropical species, ventral (top) and lateral view (bottom), respectively. **A, D**. *Aphanogmus manta* sp. nov. (holotype: BIOUG48660-B04); **B, E**. *Aphanogmus rafikii* (paratype: ZFMK-HYM-00037067); **C, F**. *Aphanogmus kisiwa* (paratype: ZFMK-HYM-00037024).

**Diagnostic comparison with similar species**. The male genitalia of *Aphanogmus manta* sp. nov. are similar to those of the Afrotropical species *Aphanogmus kisiwa* and *A. rafikii* (Salden and Peters 2023). However, *A. kisiwa* has a lower harpe/gvc index of 0.38 (0.49–0.52 in *A. manta* sp. nov.), no distinct rectangular emargination and no visible delineation (as in the holotype of *A. manta* sp. nov.) at mid-length of the lateral margin of the harpe, and the apical setae on the dorsolateral lobe are significantly longer, more than three-quarters as long as the harpe in *A. kisiwa* and around two-thirds as long as the harpe in *A. manta* sp. nov. (Fig. 4A, C, D, F). Another important difference is the shape of the flagellomeres. The flagellomeres of *A. kisiwa* are noticeably longer and thinner compared to the flagellomeres of *A. manta* sp. nov.

**Figure 5.**
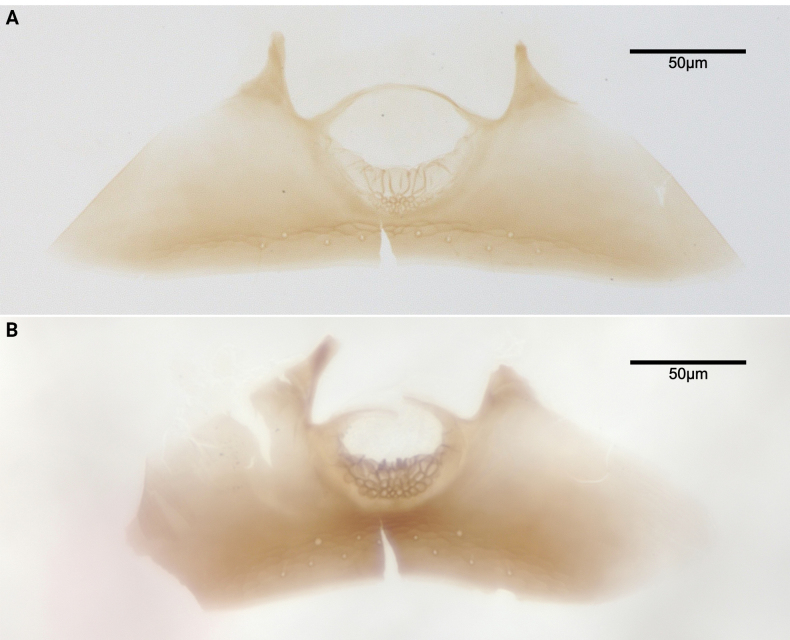
Comparison of the Waterston’s evaporatorium of *Aphanogmus manta* sp. nov. with that of the similar Afrotropical species *A. rafikii* in dorsal view. **A**. *Aphanogmus manta* sp. nov. (holotype: BIOUG48660-B04); **B**. *Aphanogmus rafikii* (paratype: ZFMK-HYM-00037068).

The male genitalia of *A. rafikii* are even more similar to those of *A. manta* sp. nov. The main difference is the ventral lobe length/harpe length of 0.44–0.49 compared to 0.37 in *A. manta* sp. nov. Additionally, the ventral lobe is more curved and tilts more lateral in *A. rafikii* (Fig. 4A, B, D, E). The Waterston’s evaporatorium of *A. rafikii* also clearly differs from that of *A. manta* sp. nov. It has a lower WE length/median T6 length of 0.59 compared to 0.67–0.72 in *A. manta* sp. nov. and a much more rounded shape. Additionally, it has a more indistinct proximomedial lamella anteriorly to the evaporatorium (Fig. 5). Note that the Waterston’s evaporatorium of *A. rafikii* was examined from the paratype (ZFMK-HYM-00037068), as the posterior metasoma of the holotype is missing.

In females, *A. manta* sp. nov. can be easily distinguished from most other species of *Aphanogmus* by the distinct spines on the seventh metasomal sternite. Within those species with sternal spines, *A. manta* sp. nov. can be distinguished from *A. liceodosriosi* sp. nov. and *A. kretschmanni* by the following characters: The Waterston’s evaporatorium in *A. liceodosriosi* sp. nov. and *A. kretschmanni* is lacking the distinct proximomedial lamella anteriorly to the evaporatorium cells which is present in *A. manta* sp. nov. Also, the WE length/median T6 length of the females of *A. manta* sp. nov. is significantly higher (0.70–0.77) compared to *A. liceodosriosi* sp. nov. (0.57–0.61) and *A. kretschmanni* (0.48–0.54). The combined shape of the bulla and the evaporatorium cells is bulbous (Fig. 3B) in *A. manta* sp. nov., while it is symmetrically (Fig. 7B) or asymmetrically oval (Fig. 10) in the other two species. The converging orientation of the tergal apodemes in *A. liceodosriosi* sp. nov. differs from the virtually parallel orientation in *A. manta* sp. nov. Additionally, the mesoscutellum of *A. manta* sp. nov. is only minimally projecting posteriorly compared to the metanoto-propodeo-metapecto-mesopectal complex in lateral view (Fig. 9B) (distinctly projecting in *A. kretschmanni* (Fig. 9A) and *A. liceodosriosi* sp. nov.). Furthermore, *A. liceodosriosi* sp. nov. has much more distinct longitudinal striations on the mesometapleuron (indistinct in *A. manta* sp. nov. and *A. kretschmanni*) (Fig. 8). The *CO1* barcode sequences clearly differ between all three species.

**Condition of the specimens**. The metasomas of all specimens are separated behind the syntergum and dissected for examination of the Waterston’s evaporatorium. Holotype: Head separated (still attached when making Fig. 2D); left middle leg missing beginning from mesofemur, right fore and middle leg missing from protrochanter and mesotrochanter; sixth metasomal tergite with a crack posteriorly; both setae on the dorsolateral lobes of the male genitalia missing. Paratypes: mesosoma of BIOUG36037-H03 separated in several pieces, parts of the legs and the antennae missing; some flagellomeres of BIOUG44884-G10 missing; head and metasoma of BIOUG45857-D01 separated, parts of the antennae and legs missing; head, meso- and metasoma of BIOUG46440-D08 damaged, parts of the antennae and legs missing, two wings missing, mesoscutellum detached from the metanoto-propodeo-metapecto-mesopectal complex; parts of the legs of CBG-A03954-G12 missing, both hind wings and one fore wing missing; sixth metasomal tergite of BIOUG36037-H03 and BIOUG46440-D08 with a crack posteriorly; additional cracks lateral to the tergal apodemes in BIOUG36037-H03; the acrotergal calyx of BIOUG44884-G10 damaged. Other material: Metasoma of BIOUG95808-F07 separated, parts of the legs missing; right hindwing and forewing of BIOUG95812-E11 missing, parts of the legs missing; mesoscutellum and mesoscutum of BIOUG95813-A08 separated from the mesosoma, head separated and parts of the legs and antennae separated or missing; mesosoma of BIOUG95813-G05 damaged, metasoma separated and legs partially separated or missing; one hind wing of BIOUG98824-E03 missing, head separated and parts of the antennae and legs missing; metasoma of BIOUG99107-G12 separated, legs partially missing; sixth metasomal tergite of BIOUG98824-E03 with a crack posteriorly; the acrotergal calyx of BIOUG95808-F07 damaged.

##### Variation.

**Figure 6.**
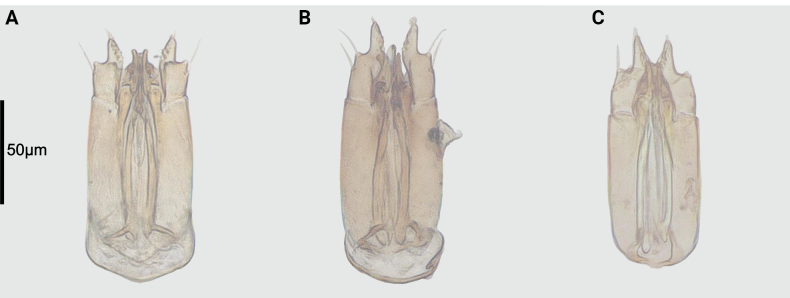
Intraspecific comparison of the male genitalia of *Aphanogmus manta* sp. nov. **A**. Male genitalia of the holotype (BIOUG48660-B04); **B**. Male genitalia of the paratype (BIOUG44884-G10); **C**. Male genitalia of an additional non-type specimen (BIOUG98824-E03).

Colouration of the holotype is overall lighter than of the male paratype (BIOUG44884-G10). The male genitalia of the holotype with visible delineation of the rectangular emargination, which cannot be seen in the male paratype and the non-type specimen (Fig. 6). The tergal apodemes of the Waterston’s evaporatorium of the male paratype more strongly diverging basally than in the holotype and the female paratypes. In female paratype BIOUG36037-H03 the tergal apodemes of the Waterston’s evaporatorium seem to be converging, but this is probably due to the cracks lateral to the apodemes.

##### Additional material.

We assigned all specimens forming the BIN BOLD:AFB8353 that are also delimited as a separate species by all other species delimitation tools based on the barcode sequence data to *Aphanogmus manta* sp. nov. because no morphological differences to the specimens of the type series were observed, neither in male or female body characters, male genitalia, or Waterston’s evaporatorium. Especially the similarity of the male genitalia would be very unusual for two different species of *Aphanogmus* (Fig. 6C). In our integrative approach, differences solely in the DNA barcode sequences do not justify delimitation and description of a separate new species. However, we did not consider the specimens of BIN BOLD:AFB8353 as paratypes either. The *CO1* barcode difference to the type series of *A. manta* sp. nov. is 6.71% to 7.76% and therefore significantly above the 3% threshold. Intraspecific variation of more than 3% is not unusual in insects (see, for example, Zhang and Bu 2022; Kärrnäs et al. 2025). Larger series of specimens might allow integrative species delimitation and possibly description of a second species. Note that including the specimens of the BIN BOLD:AFB8353 into the molecular characterisation of *A. manta* sp. nov. would not change the minimum distance to the closest species (*Aphanogmus liceodosriosi* sp. nov.).

#### Aphanogmus liceodosriosi

Taxon classificationAnimaliaHymenopteraCeraphronidae

Schneider, Salden & Peters sp. nov.

B524EEE9-549A-552C-89E7-D95D2DA4F716

https://zoobank.org/2D9741A5-80C6-498F-900D-B96B5B5B2957

Fig. 7

##### Type material.

***Holotype***: Costa Rica • 1 ♀; Guanacaste, Area de Conservacion Guanacaste, Liceo Dos Rios, LDR-1; 10.8931°N, 85.3794°W; 545 m a.s.l.; 4. Apr. 2023; Janzen D., Hallwachs W. leg.; Malaise trap; BOLD Sample ID: CBG-A33597-B12; ZFMKZFMK-Hym-00043052. ***Paratypes***: Costa Rica • 1 ♀; same collection data as holotype; 14. Mar. 2023; BOLD Sample ID: CBG-A31083-E05; CNCI • 1 ♀; same collection data as holotype; 27. Jun. 2023; BOLD Sample ID: CBG-A35817-A01; CNCI • 1 ♀; Alajuela, Upala, San Miguel de Bijagua, La Boconera, Boconera-1; 10.759°N, 85.009°W; 420 m a.s.l.; 9. Aug. 2021; Janzen D., Hallwachs W., Chacon I. leg.; Malaise trap; BOLD Sample ID: CBG-A08281-A06; CNCI. Bangladesh • 1 ♀; Chittagong, University of Chittagong, Vegetable Crop Experimental Field; 22.4685°N, 91.7808°E; 31 m a.s.l.; 7. Oct. 2014; Mazumdar S. leg.; Malaise trap; BOLD Sample ID: BIOUG24432-G07; ZFMKZFMK-Hym-00043041.

##### Diagnosis.

**Figure 7.**
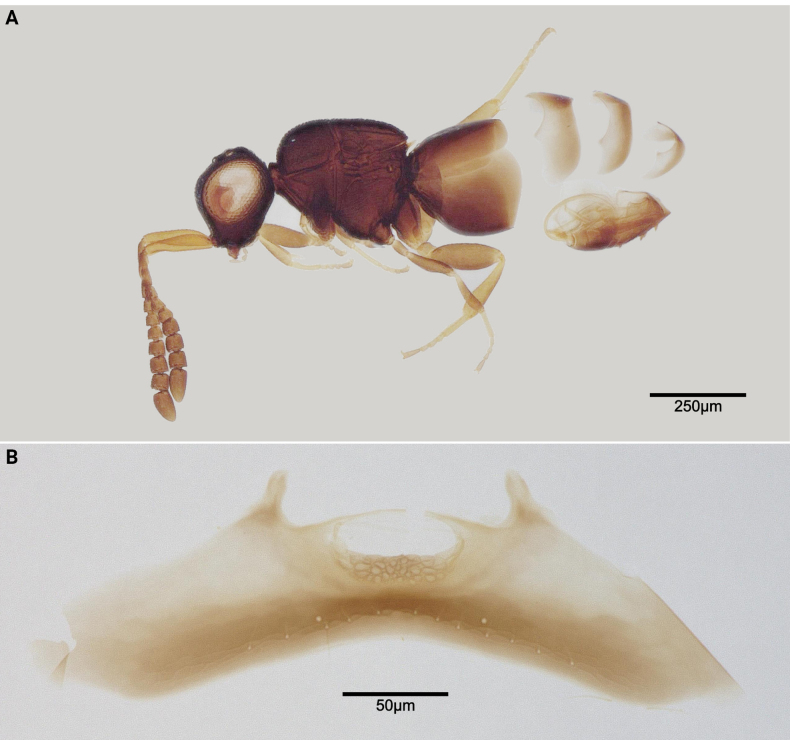
Female *Aphanogmus liceodosriosi* sp. nov. (holotype: CBG-A33597-B12). **A**. Habitus in lateral view; **B**. Waterston’s evaporatorium in dorsal view.

**Figure 8.**
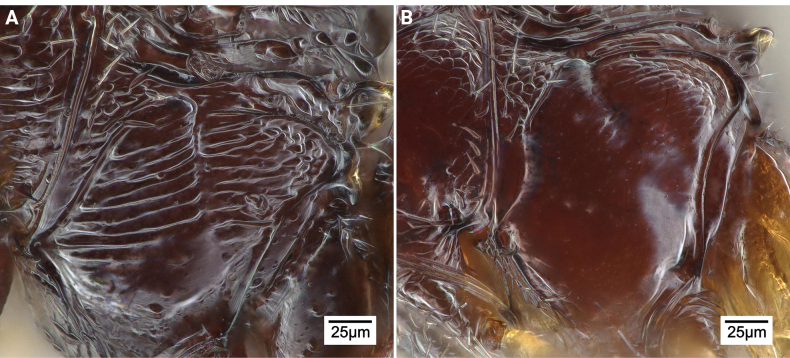
Comparison of the longitudinal striations of the mesometapleuron between *A. liceodosriosi* sp. nov. and *A. manta* sp. nov. **A**. Distinct longitudinal striations in *A. liceodosriosi* sp. nov. (female holotype CBG-A33597-B12); **B**. Indistinct longitudinal striations in *A. manta* sp. nov. (female paratype BIOUG45857-D01), similar in *A. kretschmanni*. Images taken from dry specimens, lateral view.

Female with 5–9 spines arranged in two rows on the ventral edge of the seventh metasomal sternite (Fig. 7A), mesoscutellum distinctly projecting posteriorly compared to the metanoto-propodeo-metapecto-mesopectal complex in lateral view (Fig. 7A), mesometapleuron with distinct longitudinal striations in its dorsal half (Fig. 8A), and the Waterston’s evaporatorium (WE) with WE length/median T6 length 0.57–0.61, the combined shape of the bulla and the evaporatorium symmetrically oval, one pair of campaniform sensillae present, broad and converging tergal apodemes and without distinct proximomedial lamella anteriorly to the evaporatorium (Fig. 7B).

##### Description.

**Female (*n* = 5). *Colouration***. Head dark brown; mesosoma pale to dark brown; metasoma pale brown; scape yellow to dark yellow; pedicel brown or brown-yellowish; flagellum pale brown; legs brown-yellowish; wings hyaline; wing venation pale brown; stigmal vein pale brown. ***Body length***. 0.94–1.27 mm (holotype: 0.99 mm). ***Mesosoma***. Median mesoscutal line complete. Scutoscutellar sulcus medially acutely angled, scutoscutellar sulcus and transscutal articulation not adjacent, interaxillar sulcus present. Circumscutellar carina present on mesoscutellum, ventral margin of mesoscutellum with indistinct angular carina; mesoscutellum distinctly projecting posteriorly compared to the metanoto-propodeo-metapecto-mesopectal complex in lateral view; anteromedian projection of the metanoto-propodeo-metapecto-mesopectal complex straight and tapered in lateral view, mesometapleural sulcus present in dorsal half (Fig. 7A); mesometapleuron with distinct longitudinal striations in dorsal half (Fig. 8A); metanotal-propodeal sulcus foveate (Fig. 7A). ***Metasoma***. Basal transverse carina on syntergum present; ventral edge of seventh metasomal sternite with five to nine spines in two rows (holotype: five), anteriormost two spines situated directly next to each other (Fig. 7A). ***Waterston’s evaporatorium***. Acrotergal calyx indistinctly convex; evaporatorium slightly convex posteriorly, containing 15–50 cells (26); without distinct proximomedial lamella; cell size mostly bigger than or rarely equal to setal socket size; bulla slightly convex anteriorly and posteriorly; the combined shape of the bulla and the evaporatorium cells is symmetrically oval; bulla laterally broadly rounded; WE length/median T6 length 0.57–0.61 (0.57); tergal apodemes broad and converging, mostly thickened distally; 12–17 setae in caudal setal row; one pair of campaniform sensillae present (Fig. 7B).

**Male**. Unknown.

##### *CO1* barcode.

Maximum intraspecific distance: 1.89% (*n* = 5). Minimum interspecific distance (to *Aphanogmus* sp. JS-3): 14.42%. Minimum interspecific distance to a described species: 14.77% (to *A. manta* sp. nov.).

This species corresponds to BIN BOLD:ACY0736.

##### Etymology.

The species is named in honour of the students in the Dos Rios high school (Liceo Dos Rios) who serviced weekly three Malaise traps in an adjacent rain forest for a year as part of the “Reading life to save it” project and collected the holotype and two of the paratypes of this species.

##### Distribution.

Neotropical: Costa Rica. Oriental: Bangladesh.

##### Ecology.

Unknown.

##### Remarks.

**Figure 9.**
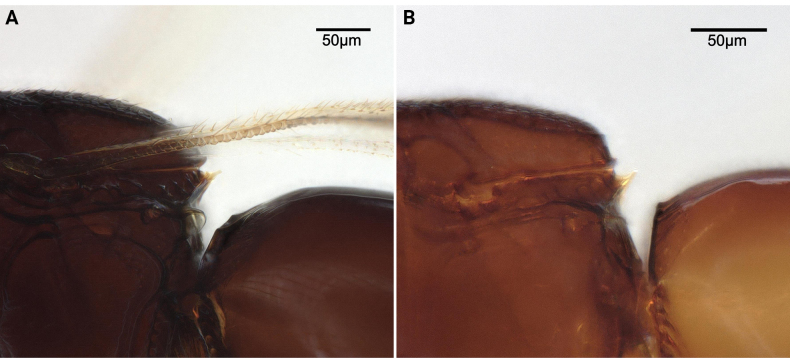
Comparison of the projection of the mesoscutellum between *Aphanogmus kretschmanni* and *A. manta* sp. nov. in lateral view. **A**. Mesoscutellum is distinctly projecting posteriorly compared to the metanoto-propodeo-metapecto-mesopectal complex in *Aphanogmus kretschmanni* (female paratype SMNS_Hym_Hym_027509); **B**. Mesoscutellum is minimally projecting posteriorly compared to the metanoto-propodeo-metapecto-mesopectal complex in *Aphanogmus manta* sp. nov. (female paratype CBG-A03954-G12).

**Figure 10.**
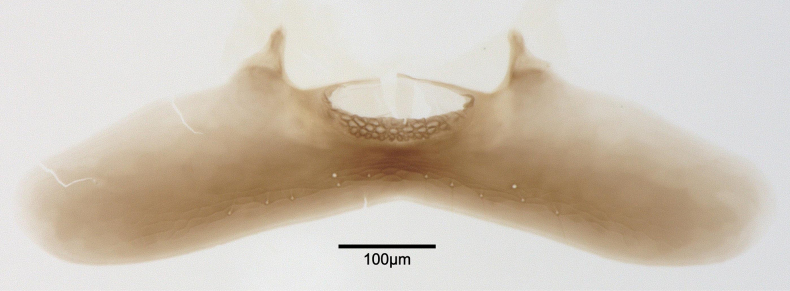
Waterston’s evaporatorium of *A. kretschmanni* (female paratype SMNS_Hym_Hym_027509).

**Diagnostic comparison with similar species**. *Aphanogmus liceodosriosi* sp. nov. can be easily distinguished from most other species of *Aphanogmus* by the distinct spines on the seventh metasomal sternite of the female. Within those species with sternal spines, the females of *A. liceodosriosi* sp. nov. can be morphologically distinguished from those of *A. kretschmanni* and *Aphanogmus manta* sp. nov. based on the distinct longitudinal striations of the mesometapleuron (indistinct in *A. kretschmanni* and *A. manta* sp. nov., Fig. 8) and characters of the Waterston’s evaporatorium: The combined shape of the bulla and the evaporatorium cells in *A. liceodosriosi* sp. nov. is different than in the other two: symmetrically oval in *A. liceodosriosi* sp. nov. (Fig. 7B), bulbous in *A. manta* sp. nov. (Fig. 3B) and asymmetrically oval in *A. kretschmanni* (Fig. 10). The tergal apodemes are converging in *A. liceodosriosi* sp. nov. (parallel in *A. kretschmanni* and *A. manta* sp. nov.). The Waterston’s evaporatorium of *A. liceodosriosi* sp. nov. is lacking the distinct proximomedial lamella that is present in *A. manta* sp. nov. and has a WE length/median T6 length of 0.57–0.61 (0.70–0.77 in *A. manta* sp. nov. and 0.48–0.54 in *A. kretschmanni*). An additional character that distinguishes *A. liceodosriosi* sp. nov. females from *A. manta* sp. nov. females is the distinctly projecting mesoscutellum compared to the metanoto-propodeo-metapecto-mesopectal complex in lateral view (only minimally projecting in *A. manta* sp. nov., Fig. 9B). The *CO1* barcode sequences clearly differ between all three species. There are no species of *Aphanogmus* described from the Neotropical realm or the Oriental realm with unknown females (Thomson 1858; Ashmead 1895; Girault 1917; Ferrière 1933; Dessart 1975, 1988, 1994; Polaszek and Dessart 1996; Evans et al. 2005; Matsuo et al. 2016; Ranjith et al. 2023), i.e., the sternal spines are sufficient to distinguish the new species from all described Neotropical and Oriental *Aphanogmus*.

**Condition of the specimens**. The metasomas of all specimens are separated behind the syntergum and dissected for examination of the Waterston’s evaporatorium. Holotype: All wings missing. Paratypes: Head of CBG-A08281-A06 separated; head and one fore wing of CBG-A35817-A01 missing, mesoscutum detached from the mesoscutellum; posterior part of metasoma, including Waterston’s evaporatorium, of CBG-A31083-E05 missing; antennae and legs of BIOUG24432-G07 damaged, mesoscutellum and mesoscutum separated from the rest of the mesosoma; legs of CBG-A31083-E05 damaged, legs of BIOUG24432-G07 partially missing; sixth metasoma tergite of CBG-A08281-A06 with a crack posteriorly; the right tergal apodeme of the Waterston’s evaporatorium of CBG-A35817-A01 folded.

##### Variation.

The holotype and the paratype BIOUG24432-G07 with an overall slightly lighter colour than the other paratypes, especially the scape and the mesosoma. Tergal apodemes of the Waterston’s evaporatorium of CBG-A35817-A01 not thickened distally.

#### Aphanogmus kretschmanni

Taxon classificationAnimaliaHymenopteraCeraphronidae

Moser, 2023

EB51BEFE-7884-5EA1-8B3F-71EA692087B5

##### Type material examined.

***Paratypes***: Germany • 1 ♀; Baden-Württemberg, Enzkreis, Königsbach-Stein, NSG 2.119 Beim Steiner Mittelberg; 48.9704°N, 8.6590°E; 181 m a.s.l.; 22 May – 5 Jun. 2019; Entomologischer Verein Krefeld e. V. 1905 leg.; Malaise trap; SMNS SMNS_Hym_Hym_027509 • 1 ♀; Baden-Württemberg, Tübingen, Hirschau, Oberes Tal, plot number 4244; 48.5050°N, 8.9935°E; 368 m a.s.l.; 17–31 Jul. 2014; Kothe T., Engelhardt M., Bartsch D. leg.; Malaise trap; ZFMK SMNS_Hym_Cer_000647.

##### Additional material.

Germany • 1 ♀ Baden-Württemberg, Tübingen, Hirschau, Oberes Tal, plot number 4244; 48.5050°N, 8.9935°E; 368 m a.s.l.; 17–31 Jul. 2014; Kothe T., Engelhardt M., Bartsch D. leg.; Malaise trap; BOLD Sample ID: SMNS_1179430; GenBank: OP722468; SMNS SMNS_Hym_Cer_000467 • 1 ♀; same collection data as for preceding; BOLD Sample ID: SMNS_1179432; GenBank: OP722465; SMNS SMNS_Hym_Cer_000468 • 1 ♀; same collection data as for preceding; 29 Aug.–12 Sep. 2014; SMNS SMNS_Hym_Cer_000440 • 1 ♀; same collection data as for preceding; SMNS SMNS_HYM_080363 • 1 ♀; same collection data as for preceding; 12–26 Sep. 2014; BOLD Sample ID: SMNS_1177257; GenBank: OP722467; SMNS SMNS_Hym_Cer_000445 • 1 ♀; same collection data as for preceding; SMNS SMNS_Hym_Cer_000446 • 1 ♀; Baden-Württemberg, Karlsruhe, Östringen, NSG 2.217 Apfelberg, plot number 9836; 49.1675°N, 8.7903°E; 181 m a.s.l.; 27 Aug.–10 Sep. 2019; Entomologischer Verein Krefeld e.V. 1905 leg.; Malaise trap; SMNS SMNS_Hym_Hym_027357 • 1 ♀; Baden-Württemberg, Enzkreis, Königsbach- Stein, NSG 2.119 Beim Steiner Mittelberg; 48.9704°N, 8.6590°E; 181 m a.s.l.; 28 Aug.–11 Sep. 2019; Entomologischer Verein Krefeld e.V. 1905 leg.; Malaise trap; SMNS SMNS_Hym_Hym_027685 • 1 ♀; same collection data as for preceding; SMNS SMNS_HYM_080362.

##### Diagnosis.

Female with 5–10 spines arranged in two rows on the ventral edge of the seventh metasomal sternite, mesoscutellum distinctly projecting posteriorly compared to the metanoto-propodeo-metapecto-mesopectal complex in lateral view (Fig. 9A), mesometapleuron with indistinct longitudinal striations in its dorsal half, and the Waterston’s evaporatorium (WE) with WE length/median T6 length 0.48–0.54, the combined shape of the bulla and the evaporatorium asymmetrically oval, one pair of campaniform sensillae usually present, virtually parallel tergal apodemes and without distinct proximomedial lamella anteriorly to the evaporatorium (Fig. 10).

##### Description.

**Female (*n* = 11). *Colouration***. Head and mesosoma dark brown to black; metasoma brown to black; scape and pedicel brown to dark brown or brown-yellowish; flagellum brown to dark brown; legs pale brown to brown-yellowish; wings hyaline; wing venation pale brown; stigmal vein pale brown. ***Body length***. 0.7–1.1 mm (Moser et al. 2023). ***Mesosoma***. Median mesoscutal line complete. Scutoscutellar sulcus medially acutely angled, scutoscutellar sulcus and transscutal articulation not adjacent, interaxillar sulcus present. Circumscutellar carina present on mesoscutellum, ventral margin of mesoscutellum with distinct angular carina; mesoscutellum distinctly projecting posteriorly compared to the metanoto-propodeo-metapecto-mesopectal complex in lateral view (Fig. 9A); anteromedian projection of the metanoto-propodeo-metapecto-mesopectal complex straight and tapered in lateral view, mesometapleural sulcus present in dorsal half; mesometapleuron with indistinct longitudinal striations in dorsal half; metanotal-propodeal sulcus foveate. ***Metasoma***. Basal transverse carina on syntergum present; ventral edge of seventh metasomal sternite with five to ten spines in two rows, anteriormost two spines situated directly next to each other. ***Waterston’s evaporatorium***. Acrotergal calyx straight to slightly convex; evaporatorium slightly convex posteriorly, containing 21–36 cells; without distinct proximomedial lamella; cell size mostly bigger than or sometimes equal to setal socket size; bulla straight to slightly convex anteriorly and slightly convex posteriorly; the combined shape of the bulla and the evaporatorium cells is asymmetrically oval; WE length/median T6 length 0.48–0.54; tergal apodemes virtually parallel, generally narrow; 9 to 11 setae in caudal setal row; one pair of campaniform sensillae usually present (see variation below; Fig. 10).

##### Remarks.

**Diagnostic comparison with similar species**. The distinct spines on the seventh metasomal sternite in female specimens distinguish *A. kretschmanni* from most other species of *Aphanogmus*. Morphological characters that distinguish the species from the other species that share this trait are the indistinct longitudinal striations of the mesometapleuron (distinct in *A. liceodosriosi* sp. nov., Fig. 8A) and the distinctly projecting mesoscutellum compared to the metanoto-propodeo-metapecto-mesopectal complex in lateral view (Fig. 9A) (only minimally projecting in *A. manta* sp. nov., Fig. 9B). There are also characters of the Waterson’s evaporatorium that distinguish *A. kretschmanni* from the other two species: The combined shape of the bulla and the evaporatorium cells is asymmetrically oval (Fig. 10) (symmetrical oval in *A. liceodosriosi* sp. nov., Fig. 7B and bulbous in *A. manta* sp. nov., Fig. 3B), the absence of the distinct proximomedial lamella (present in *A. manta* sp. nov.), the virtually parallel orientation of the tergal apodemes (converging in *A. liceodosriosi* sp. nov.) and the differing values for the WE length/median T6 length (*A. kretschmanni*: 0.48–0.54; *A. liceodosriosi* sp. nov.: 0.57–0.61; *A. manta* sp. nov.: 0.70–0.77). The *CO1* barcode sequences clearly differ between all three species.

**Condition of the specimens**. The metasomas of all specimens are separated behind the syntergum and dissected for examination of the Waterston’s evaporatorium. Paratypes: SMNS_Hym_Cer_000647 in immaculate condition except for damage caused during dissection; acrotergal calyx of the Waterston’s evaporatorium of SMNS_Hym_Hym_027509 damaged, the sixth metasomal tergite with two cracks. Other Material: SMNS_HYM_080363 in immaculate condition except for damage caused during dissection; the sixth metasomal tergite and acrotergal calyx of the Waterston’s evaporatorium damaged in SMNS_Hym_Cer_000440 and SMNS_HYM_080362; left hind wing and parts of the legs of SMNS_Hym_Hym_027685 missing, sixth metasomal tergite, one tergal apodeme and acrotergal calyx of the Waterston’s evaporatorium damaged; mesoscutellum of SMNS_Hym_Cer_000467 detached from the metanoto-propodeo-metapecto-mesopectal complex, parts of the legs missing and parts of the antennae separated or missing; parts of the legs and left hind wing of SMNS_Hym_Cer_000468 missing, acrotergal calyx of the Waterston’s evaporatorium damaged; mesoscutellum of SMNS_Hym_Cer_000445 detached from the metanoto-propodeo-metapecto-mesopectal complex, head, parts of the legs, both hind wings and left fore wing missing, acrotergal calyx of the Waterston’s evaporatorium damaged; left hind wing of SMNS_Hym_Hym_027357 separated, acrotergal calyx and one tergal apodeme of the Waterston’s evaporatorium damaged; metasoma of SMNS_Hym_Cer_000446 separated, some flagellomeres missing, left fore wing damaged, right fore wing and both hind wings missing. The sixth metasomal tergite including the Waterston’s evaporatorium was destroyed during dissection in SMNS_HYM_080363, SMNS_Hym_Cer_000467, SMNS_Hym_Cer_000446 and the paratype SMNS_Hym_Cer_000647.

##### Variation.

Colouration of SMNS_Hym_Cer_000445, SMNS_Hym_Cer_000446, SMNS_Hym_Cer_000467 and SMNS_HYM_080363 is overall lighter than for the rest of the examined material. The mesoscutellum of the paratype SMNS_Hym_Cer_000647 has a less distinct angular carina than the other specimens.

Contrary to Moser et al. (2023), we found campaniform sensillae on the Waterston’s evaporatorium bearing tergite in *Aphanogmus kretschmanni* (Fig. 10). Specimens SMNS_Hym_Cer_000445, SMNS_Hym_Hym_027685 and SMNS_Hym_Cer_000468 only have one campaniform sensilla, all other examined specimens have a pair of campaniform sensillae. Among all species examined herein, having a pair of campaniform sensillae is the most prevalent condition. In some cases, only one sensilla is present; BIOUG15337-B09 (*A.* sp. JS-1) has three such sensillae. Only three specimens show a complete absence of campaniform sensillae: BIOUG15074-G11 (BIN BOLD:ACP1659), CBG-A01502-B05 (*A.* sp. JS-1) and CBG-A41086-B05 (*A.* sp. JS-3). This leads to the conclusion that the presence of campaniform sensillae is subject to intraspecific variation, which can also explain the absence of campaniform sensillae in the *A. kretschmanni* specimen examined by Moser et al. (2023).

### Key to *Aphanogmus* species with spiny metasomal sternite

Currently, males are known only from one of the species (*A. manta* sp. nov.). It can be diagnosed against all other described *Aphanogmus* species (see respective taxonomic treatment) and we refrain from including a key to *Aphanogmus* males herein.

**Table:** 

1	Mesometapleuron with distinct longitudinal striations (Fig. 8A); Waterston’s evaporatorium with combined shape of the bulla and evaporatorium cells symmetrically oval and broad, converging tergal apodemes (Fig. 7B)	***A. liceodosriosi* sp. nov**.
–	Mesometapleuron with indistinct longitudinal striations (Fig. 8B); Waterston’s evaporatorium with differently shaped combined bulla and evaporatorium cells (as in Figs 3B, 10) and broad or narrow but virtually parallel tergal apodemes	**2**
2	Mesoscutellum minimally projecting posteriorly compared to the metanoto-propodeo-metapecto-mesopectal complex in lateral view (Fig. 9B); Waterston’s evaporatorium with a WE length/median T6 length of 0.70–0.77 and a distinct proximomedial lamella, that extends far beyond the evaporatorium cells anteriorly (Fig. 3B)	***A. manta* sp. nov**.
–	Mesoscutellum distinctly projecting posteriorly compared to the metanoto-propodeo-metapecto-mesopectal complex in lateral view (Fig. 9A); Waterston’s evaporatorium with a WE length/median T6 length of 0.48–0.54 and without such a distinct proximomedial lamella (Fig. 10)	***A. kretschmanni* Moser, 2023**

### Assignment of examined species to a species group

Moser et al. (2023) placed *A. kretschmanni* in the *A. fumipennis* species group (Evans et al. 2005), based on a complete mesoscutal median sulcus (=median mesoscutal line) and the presence of a gastral basal carina (=basal transverse carina on syntergum) (both characters also present in all species examined here). However, they already mentioned that *A. kretschmanni* is easily distinguishable from *A. fumipennis* based on the absence of the prominent tufts of dense setae along the outer margin of the hind coxae. Salden et al. (2026a) re-defined the *A. fumipennis* species group using the “posterior mesosomal comb” as a main diagnostic character which is clearly absent in all the specimens and species examined here. Moser et al. (2023) also discuss similarities with *Elysoceraphron* because of the missing campaniform sensillae of the Waterston’s evaporatorium. As shown in this study, this trait can vary and *A. kretschmanni* usually has campaniform sensillae. Thus, the statement of Ulmer et al. (2021) that all genera of Ceraphronidae except *Elysoceraphron* (and *Synarsis* which has two pairs) have a pair of campaniform sensillae should be amended to state that this is “usually” the case. Moser et al. (2023) also discuss morphological characters of *A. kretschmanni* that are matching with the *A. hakonensis* species group (Polaszek and Dessart 1996), for example, the prominent anteromedian projection of the metanoto-propodeo-metapecto-mesopectal complex and the longitudinal striations of the mesometapleuron (Polaszek and Dessart 1996). However, there are also significant differences between the *hakonensis* group and the species examined herein, for example the mesometapleural sulcus extending halfway across the mesometapleuron (no mesometapleural sulcus in the *hakonensis* species group) and the conspicuous circumscutellar carina on the mesoscutellum (not circumscutellar carina in *hakonensis* species group). We here assign the species, based on the bilobed harpes of the male genitalia of *A. manta* sp. nov. to the *Aphanogmus nigripes*-group tentatively defined by Mikó et al. (2013).

### Taxa in open nomenclature

In the following, we describe but not formally name three additional species with spiny sternite females. They differ from each other and all described species by their DNA barcode sequences (Fig. 1) but lack morphological diagnostic characters also in their Waterston’s evaporatoria and their male genitalia (no males available). We provide details on material examined, morphological characters, DNA barcodes, and condition of the specimens to facilitate future taxonomic work if more material or other diagnostic characters become available.

#### Aphanogmus sp. JS-1

Taxon classificationAnimaliaHymenopteraCeraphronidae

DBE93967-D5A9-57FF-9E09-81D4517CBB83

Fig. 11

##### Material.

Pakistan • 1 ♀; Islamabad, Pakistan Museum of Natural History; 33.6863°N, 73.0763°E; 527 m a.s.l.; 29. Nov. 2012; Rafique M., Abbas Q. leg.; Malaise trap; BOLD Sample ID: BIOUG15337-B09; BIOUG • 1 ♀; Punjab, Dera Ghazi Khan; 30.043°N, 70.623°E; 126 m a.s.l.; 22. May 2020; Tufail M. leg.; Malaise trap; BOLD Sample ID: CBG-A00525-E08; BIOUG • 1 ♀; same collection data as for preceding; 24. Apr. 2020; BOLD Sample ID: CBG-A01502-B05; BIOUG • 1 ♀; Punjab, Bhakkar District, Bhakkar; 31.601°N, 71.061°E; 172 m a.s.l.; 27. June 2020; Khan A. leg.; Malaise trap; BOLD Sample ID: CBG-A21388-A12; BIOUG.

##### Note.

There are three additional sequences available on BOLD assigned to BIN BOLD:ACP4134 that could not be located nor examined morphologically and that were not included in the DNA barcode analyses but may represent additional records of *Aphanogmus* sp. JS-1. BOLD Sample IDs: CBG-A01501-C09, CBG-A62562-B11, CBG-A21319-E08.

##### Description.

**Figure 11.**
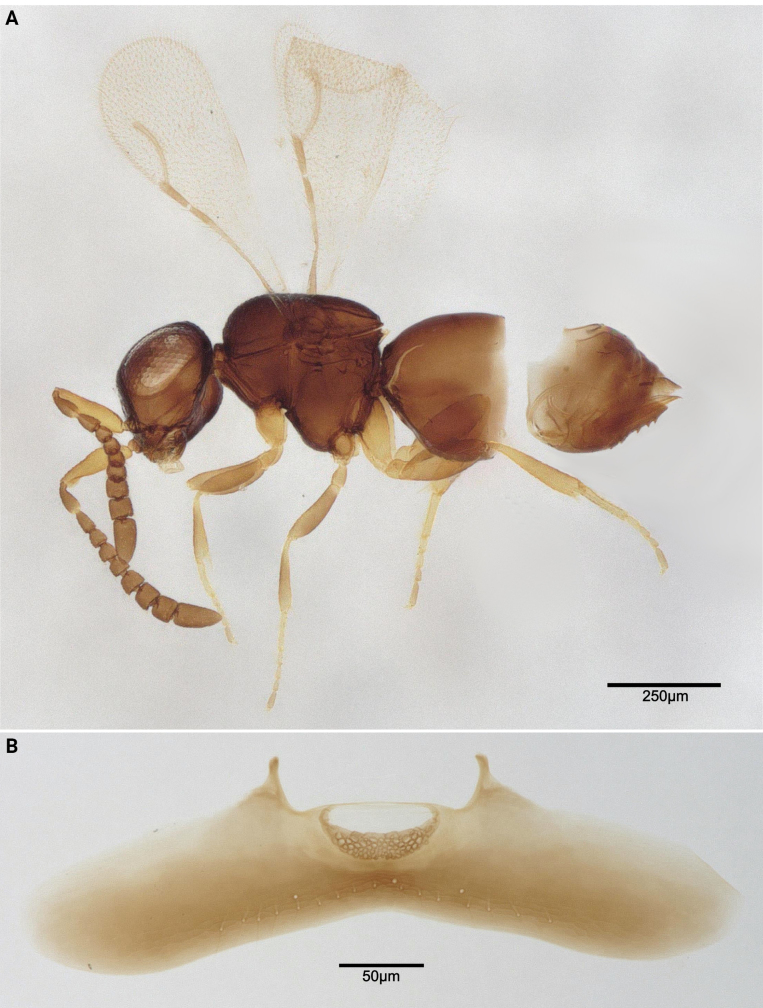
Female *Aphanogmus* sp. JS-1. **A**. Habitus in lateral view (CBG-A01502-B05); **B**. Waterston’s evaporatorium in dorsal view (BIOUG15337-B09).

**Female (*n* = 4). *Colouration***. Head pale to dark brown; mesosoma pale to dark brown; metasoma pale brown; scape yellow; pedicel brown-yellowish; flagellum pale brown; legs pale yellow and transparent to brown-yellowish; wings hyaline; wing venation pale brown; stigmal vein pale brown. ***Body length***. 0.92–1.10 mm. ***Mesosoma***. Median mesoscutal line complete. Scutoscutellar sulcus medially acutely angled, scutoscutellar sulcus and transscutal articulation not adjacent, interaxillar sulcus present. Circumscutellar carina present on mesoscutellum, ventral margin of mesoscutellum with distinct angular carina; mesoscutellum distinctly projecting posteriorly compared to the metanoto-propodeo-metapecto-mesopectal complex in lateral view; anteromedian projection of the metanoto-propodeo-metapecto-mesopectal complex straight and tapered in lateral view, mesometapleural sulcus present in dorsal half; mesometapleuron with indistinct longitudinal striations in dorsal half; metanotal-propodeal sulcus foveate (Fig. 11A). ***Metasoma***. Basal transverse carina on syntergum present; ventral edge of seventh metasomal sternite with five to seven spines in two rows, anteriormost two spines situated directly next to each other (Fig. 11A). ***Waterston’s evaporatorium***. Acrotergal calyx straight; evaporatorium slightly convex posteriorly, containing 8 to 60 cells; without distinct proximomedial lamella; cell size mostly bigger than or sometimes equal to setal socket size; bulla straight anteriorly and slightly convex posteriorly; the combined shape of the bulla and the evaporatorium cells is asymmetrically oval; WE length/median T6 length 0.44–0.52; tergal apodemes narrow and diverging, sometimes thickened distally; 10 to 16 setae in caudal setal row; one pair of campaniform sensillae usually present (see variation under *A. kretschmanni*; Fig. 11B).

**Male**. Unknown.

##### *CO1* barcode.

Maximum intraspecific distance: 1.87% (*n* = 4). Minimum interspecific distance (to *Aphanogmus kretschmanni*): 9.55%.

This species corresponds to BIN BOLD:ACP4134.

##### Distribution.

Palaearctic: Pakistan.

##### Ecology.

Unknown.

##### Remarks.

**Condition of the specimens**. The metasomas of all specimens are separated behind the syntergum and dissected for examination of the Waterston’s evaporatorium. Parts of the legs and at least one wing missing in all specimens; head of CBG-A00525-E08 separated and antennae partially missing; mesoscutellum of CBG-A21388-A12 detached from the metanoto-propodeo-metapecto-mesopectal complex, head separated and antennae partially missing; head of CBG-A21388-A12 divided into two pieces and both forelegs and a part of the propleuron separated; one hind leg separated beginning from the metatrochanter; acrotergal calyx of Waterston’s evaporatorium of CBG-A01502-B05 damaged; acrotergal calyx of Waterston’s evaporatorium of CBG-A21388-A12 damaged; mesoscutellum of BIOUG15337-B09 detached from the metanoto-propodeo-metapecto-mesopectal complex.

##### Variation.

Bulla of Waterston’s evaporatorium in CBG-A21388-A12 more rounded anteriorly than in the other specimens, possibly due to the damaged acrotergal calyx.

#### Aphanogmus

Taxon classificationAnimaliaHymenopteraCeraphronidae

sp. JS-2

791460DB-3C4F-5AF9-BC52-9A47DE985989

Fig. 12

##### Material.

Australia • 1 ♀; Queensland, Hemit Park; 19.283°S, 146.801°E; 10 m a.s.l.; 7. Aug. 2017; Cocks G. leg.; Malaise trap; BOLD Sample ID: BIOUG41413-C05; BIOUG • 3 ♀♀; same collection data as for preceding; 24. Jul. 2017; BOLD Sample IDs: BIOUG41478-G01, BIOUG41479-C12, BIOUG41480-A05; BIOUG • 3 ♀♀; same collection data as for preceding; 21. Aug. 2017; BOLD Sample IDs: BIOUG41494-G06, BIOUG41495-F06, BIOUG41561-C07; BIOUG • 1 ♀; same collection data as for preceding; 4. Sep. 2017; BOLD Sample ID: BIOUG41571-C02 • 2 ♀♀; same collection data as for preceding; 18. Sep. 2017; BOLD Sample IDs: BIOUG41499-G12, BIOUG41503-C05; BIOUG.

##### Note.

There is one additional sequence available on BOLD assigned to BIN BOLD:ADU4363 that could not be located and not examined morphologically and that was not included in the DNA barcode analyses but may represent an additional record of *Aphanogmus* sp. JS-2. BOLD Sample ID: CBG-A55750-H05.

##### Description.

**Figure 12.**
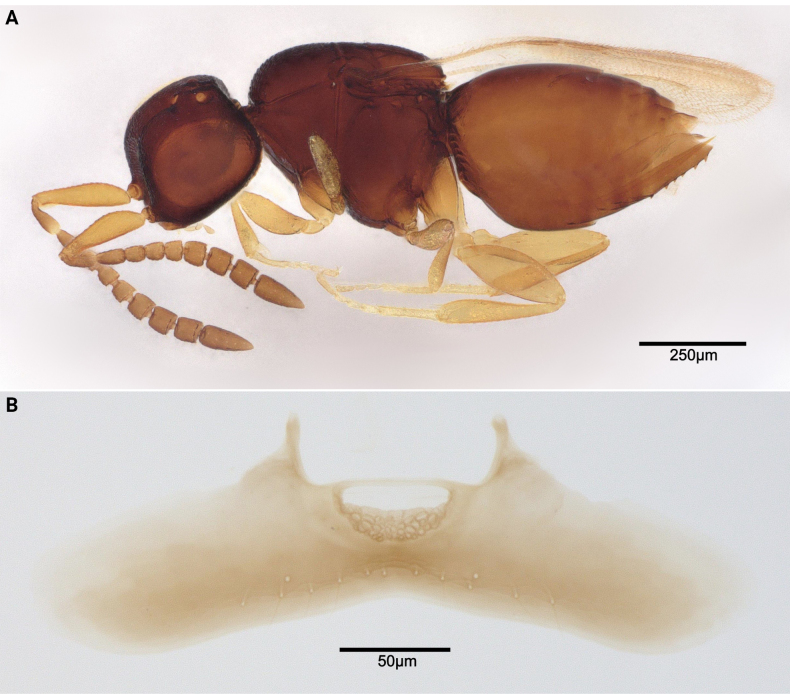
Female *Aphanogmus* sp. JS-2. **A**. Habitus in lateral view (BIOUG41571-C02); **B**. Waterston’s evaporatorium in dorsal view (BIOUG41503-C05).

**Female (*n* = 10). *Colouration***. Head pale to dark brown; mesosoma pale to dark brown; metasoma pale brown; scape yellow to dark yellow; pedicel yellow-brownish or brown; flagellum pale brown; legs yellow-transparent to pale brown; wings hyaline; wing venation pale brown; stigmal vein pale brown. ***Body length***. 0.77–1.10 mm. ***Mesosoma***. Median mesoscutal line complete. Scutoscutellar sulcus medially acutely angled, scutoscutellar sulcus and transscutal articulation not adjacent, interaxillar sulcus present. Circumscutellar carina present on mesoscutellum, ventral margin of mesoscutellum with distinct angular carina; mesoscutellum somewhat projecting posteriorly compared to the metanoto-propodeo-metapecto-mesopectal complex in lateral view; anteromedian projection of the metanoto-propodeo-metapecto-mesopectal complex straight and tapered in lateral view, mesometapleural sulcus present in dorsal half; mesometapleuron with indistinct longitudinal striations in dorsal half; metanotal-propodeal sulcus foveate (Fig. 12A). ***Metasoma***. Basal transverse carina on syntergum present; ventral edge of seventh metasomal sternite with four to eight spines in two rows, anteriormost two spines situated directly next to each other (Figs 12A, 17). ***Waterston’s evaporatorium***. Acrotergal calyx straight; evaporatorium slightly convex posteriorly, containing 15–30 cells; without distinct proximomedial lamella; cell size bigger than or equal to setal socket size; bulla straight anteriorly and slightly convex posteriorly; the combined shape of the bulla and the evaporatorium cells is asymmetrically oval; WE length/median T6 length 0.44–0.59; tergal apodemes generally parallel; 7–13 setae in caudal setal row; one pair of campaniform sensillae present (Fig. 12B).

**Male**. Unknown.

##### *CO1* barcode.

Maximum intraspecific distance: 0.78% (*n* = 10). Minimum interspecific distance (to *Aphanogmus kretschmanni*): 7.17%.

This species corresponds to BIN BOLD:ADU4363.

##### Distribution.

Australasian: Australia.

##### Ecology.

Unknown.

##### Remarks.

**Condition of the specimens**. The metasomas of all specimens are separated behind the syntergum and dissected for examination of the Waterston’s evaporatorium. Head of BIOUG41413-C05, BIOUG41499-G12 and BIOUG41478-G01 separated and legs and antennae partially missing; two wings of BIOUG41478-G01 missing; head of BIOUG41479-C12 separated and damaged, metasoma and Waterston’s evaporatorium missing, legs and antennae partially missing; head of BIOUG41480-A05 separated and metasoma including Waterston’s evaporatorium missing, parts of the legs separated and missing; head of BIOUG41494-G06 separated; head of BIOUG41495-F06 separated, legs partially missing, one hind leg separated; head of BIOUG41503-C05 separated, metasoma damaged and legs partially missing; head and metasoma of BIOUG41561-C07 separated, legs and the antennae partially separated or missing, mesoscutellum detached from the metanoto-propodeo-metapecto-mesopectal complex; legs of BIOUG41571-C02 partially missing; sixth metasomal tergite of BIOUG41571-C02 with a crack posteriorly; the acrotergal calyx of the Waterston’s evaporatorium of BIOUG41494-G06, BIOUG41495-F06, BIOUG41499-G12 and BIOUG41571-C02 damaged; the left tergal apodeme of the Waterston’s evaporatorium of BIOUG41495-F06 folded; the tergal apodemes of the Waterston’s evaporatorium of BIOUG41499-G12 separated.

##### Variation.

The evaporatorium cells + the bulla of the Waterston’s evaporatorium of BIOUG41571-C02 slightly more rounded than in most other specimens. The tergal apodemes of the Waterston’s evaporatorium of BIOUG41413-C05 slightly converging and of BIOUG41561-C07 slightly diverging.

#### Aphanogmus

Taxon classificationAnimaliaHymenopteraCeraphronidae

sp. JS-3

D1A0DD37-0FBE-55A6-84C2-D291E1F15D13

Fig. 13

##### Material.

Pakistan • 1 ♀; Azad Kashmir, Poonch District, Rawalakot, University of Poonch; 33.8474°N, 73.7744°E; 1646 m a.s.l.; 13. Aug. 2018; Khan N.N. leg.; Malaise trap; BOLD Sample ID: CBG-A40805-B01; BIOUG. • 1 ♀; same collection data as for preceding; 15. Jul. 2018; BOLD Sample ID: CBG-A41086-B05; BIOUG. • 1 ♀; same collection data as for preceding; 27. Aug. 2018; BOLD Sample ID: CBG-A41127-A09; BIOUG. • 1 ♀; same collection data as for preceding; 24. Sep. 2018; BOLD Sample ID: CBG-A41155-D12; BIOUG.

##### Note.

There are two additional sequences available on BOLD assigned to BIN BOLD:AFF8142 that could not be located and not examined morphologically and that were not included in the DNA barcode analyses but may represent additional records of *Aphanogmus* sp. JS-3. BOLD Sample IDs: BIOUG97698-E04, CBG-A26152-G12.

##### Description.

**Figure 13.**
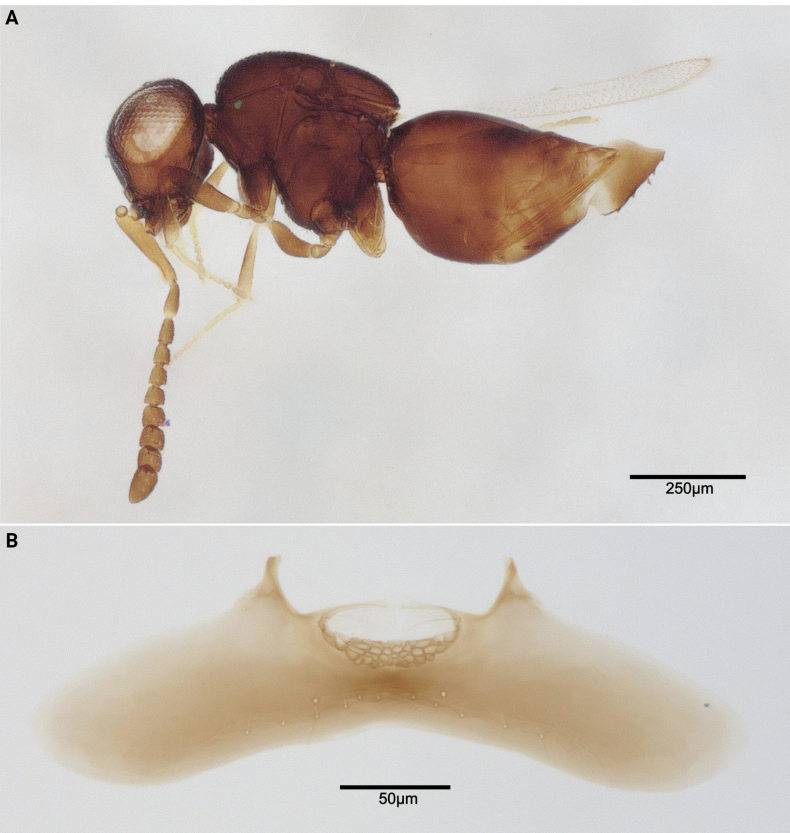
Female *Aphanogmus* sp. JS-3. **A**. Habitus in lateral view (CBG-A40805-B01); **B**. Waterston’s evaporatorium in dorsal view (CBG-A40805-B01).

**Female (*n* = 4). *Colouration***. Head pale to dark brown; mesosoma pale to dark brown; metasoma pale brown; scape yellow to dark yellow; pedicel yellow-brownish; flagellum pale brown; legs yellow-transparent to yellow-brownish; wings hyaline; wing venation pale brown; stigmal vein pale brown. ***Body length***. 0.92–1.05 mm. ***Mesosoma***. Median mesoscutal line complete. Scutoscutellar sulcus medially acutely angled, scutoscutellar sulcus and transscutal articulation not adjacent, interaxillar sulcus present. Circumscutellar carina present on mesoscutellum, ventral margin of mesoscutellum with distinct angular carina; mesoscutellum distinctly projecting posteriorly compared to the metanoto-propodeo-metapecto-mesopectal complex in lateral view; anteromedian projection of the metanoto-propodeo-metapecto-mesopectal complex straight and tapered in lateral view, mesometapleural sulcus present in dorsal half; mesometapleuron with indistinct longitudinal striations in dorsal half; metanotal-propodeal sulcus foveate (Fig. 13A). ***Metasoma***. Basal transverse carina on syntergum present; ventral edge of seventh metasomal sternite with five to eleven spines in two rows, anteriormost two spines situated directly next to each other (Fig. 13A). ***Waterston’s evaporatorium***. Acrotergal calyx straight; evaporatorium slightly convex posteriorly, containing 20 to 30 cells; without distinct proximomedial lamella; cell size bigger than setal socket size; bulla straight anteriorly and slightly convex posteriorly; the combined shape of the bulla and the evaporatorium cells is asymmetrically oval; WE length/median T6 length 0.49–0.56; tergal apodemes slightly diverging, sometimes thickened distally and bent medially; 11 setae in caudal setal row; one pair of campaniform sensillae usually present (see variation under *A. kretschmanni*; Fig. 13B).

**Male**. Unknown.

##### *CO1* barcode.

Maximum intraspecific distance: 1.06% (*n* = 4). Minimum interspecific distance (to *Aphanogmus kretschmanni*): 9.18%.

This species corresponds to BIN BOLD:AFF8142.

##### Distribution.

Palearctic: Pakistan.

##### Ecology.

Unknown.

##### Remarks.

**Condition of the specimens**. The metasomas of all specimens are separated behind the syntergum and dissected for examination of the Waterston’s evaporatorium. Antennae of CBG-A40805-B01 separated, both fore wings missing and parts of the legs missing; head of CBG-A41086-B05 separated, one hind leg partially missing and mesoscutellum detached from the metanoto-propodeo-metapecto-mesopectal complex; mesoscutellum of CBG-A41127-A09 detached from the metanoto-propodeo-metapecto-mesopectal complex, one fore wing and head separated; head of CBG-A41155-D12 missing and left hind leg separated; acrotergal calyx of the Waterston’s evaporatorium of CBG-A40805-B01, CBG-A41127-A09 and CBG-A41155-D12 damaged.

##### Variation.

Tips of the tergal apodemes of the Waterston’s evaporatorium of CBG-A41086-B05 and CBG-A41155-D12 medially tilted.

### Additional specimens not assigned to species

Below, we mention three additional specimens with sternal spines which each form a separate BIN and are combined with or delimited from the other species in various ways by the other species delimitation tools (see results of molecular analyses and Fig. 1). Because all BINs/putative species include only one (female) specimen, intraspecific variation cannot be assessed. We do not provide morphological descriptions but list here some comparative morphological characters and provide images that might be useful for future taxonomic work.

**Material examined**. Malaysia • 1 ♀; Selangor, Gombak Field Studies Centre; 3.317°N, 101.75°E; 17. Aug. 2012; Wilson J. leg.; Malaise trap; BOLD Sample ID: BIOUG15074-G11 (BIN BOLD:ACP1659); BIOUG. Thailand • 1 ♀; Nan, Pua, Phu Ka, Doi Phu Kha National Park (DPKNP), stargazing field, Doi Phukha 3; 19.205°N, 101.081°E; 1356 m a.s.l.; 7. Oct. 2022; Butcher B. leg.; Malaise trap; BOLD Sample ID: CBG-A00991-G09 (BIN BOLD:AFS2572); BIOUG. Russia • 1 ♀; Primorskiy Kray, Kiparisovo (Tazyozhnyi); 43.4858°N, 131.975°E; 25. Jul. 2015; Voronkov A. leg.; Malaise trap; BOLD Sample ID: BIOUG27197-B04 (BIN BOLD:AFS2573); BIOUG.

**Figure 14.**
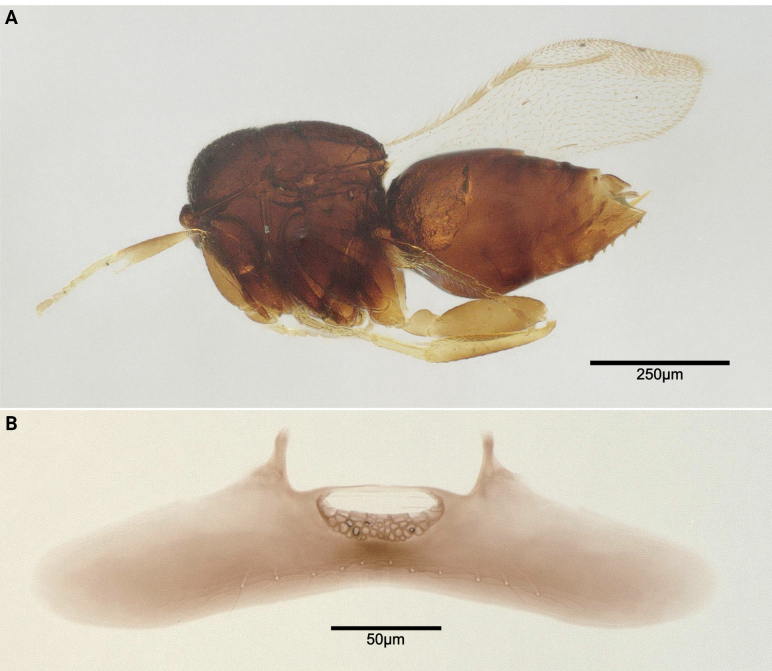
BIOUG15074-G11 (female). **A**. Habitus in lateral view; **B**. Waterston’s evaporatorium in dorsal view.

**Figure 15.**
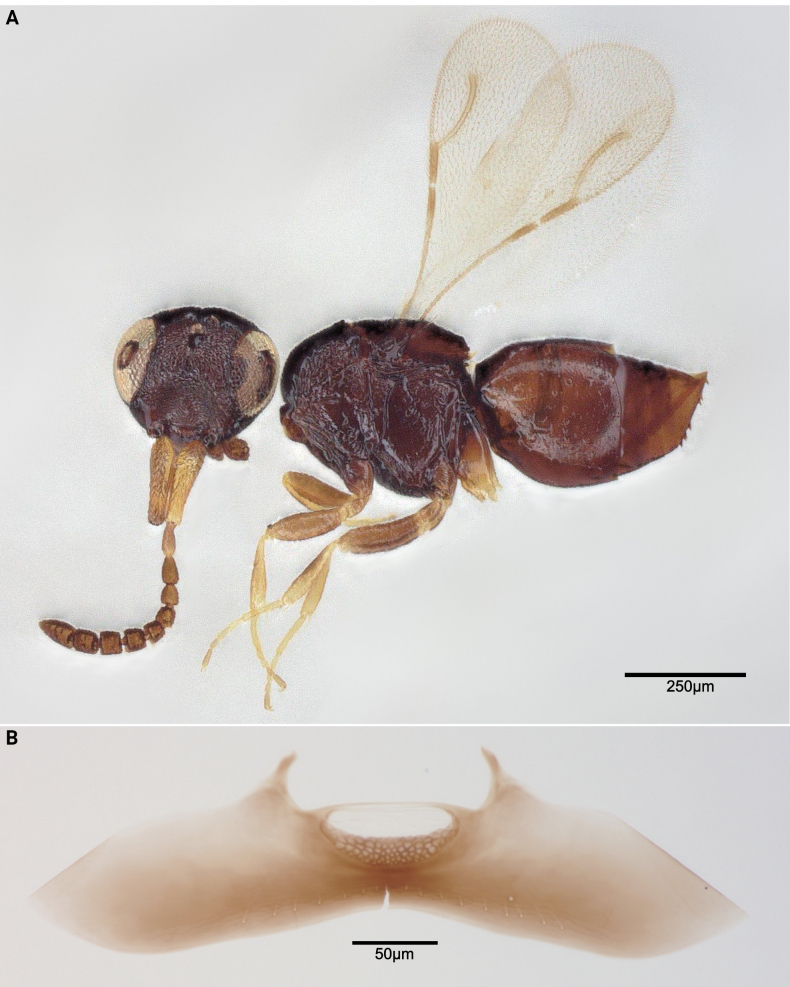
CBG-A00991-G09 (female). **A**. Habitus in lateral view (dry specimen); **B**. Waterston’s evaporatorium in dorsal view.

**Figure 16.**
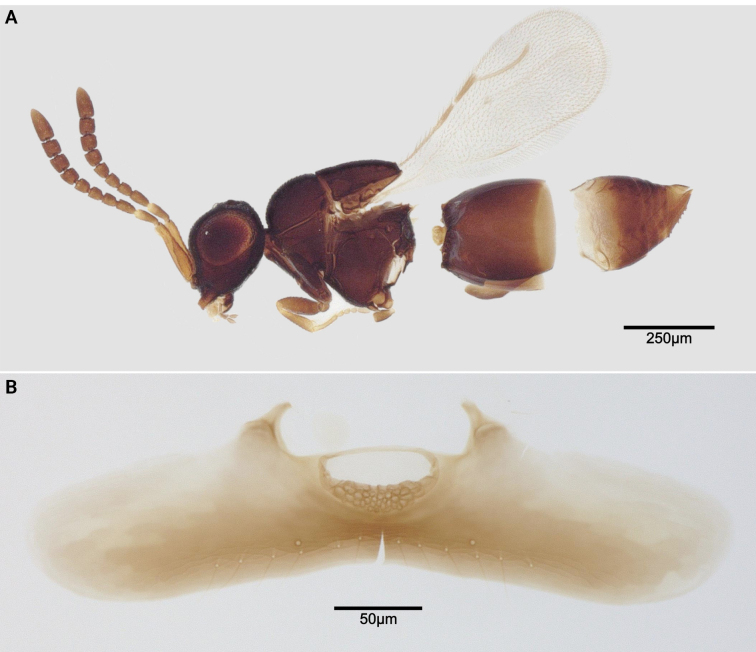
BIOUG27197-B04 (female). **A**. Habitus in lateral view; **B**. Waterston’s evaporatorium in dorsal view.

**Comparison with described species and species in open nomenclature**. The longitudinal striations of the mesometapleuron of all three specimens are indistinct (distinct in *Aphanogmus liceodosriosi* sp. nov., Fig. 8A) (Figs 14A, 15A, 16A). The mesoscutellum is distinctly projecting posteriorly compared to the metanoto-propodeo-metapecto-mesopectal complex in lateral view in BIOUG15074-G11 and CBG-A00991-G09 (minimally projecting in *Aphanogmus manta* sp. nov., Fig. 9B) (Figs 14A, 15A) (undefinable in BIOUG27197-B04 because of damaged mesosoma, Fig. 16A). The Waterston’s evaporatorium of BIOUG15074-G11 is similar to that of *Aphanogmus* sp. JS-1 or JS-2 (Fig. 14A). The Waterston’s evaporatoria of CBG-A00991-G09 and BIOUG27197-B04 are similar to each other except for the tergal apodemes more bulging in BIOUG27197-B04 (Fig. 16B) than in CBG-A00991-G09 (Fig. 15B), but are different from those of the remaining examined species. The acrotergal calyx is straight and the bulla is convex posteriorly (Figs 15B, 16B), resembling *Aphanogmus* sp. JS-1 or JS-3. The tergal apodemes are converging, resembling *A. liceodosriosi* sp. nov., but tergal apodemes are less broad than in *A. liceodosriosi* sp. nov. and the combined shape of the bulla and the evaporatorium cells is asymmetrically oval (symmetrically oval in *A. liceodosriosi* sp. nov., Fig. 7B).

Regarding the *CO1* barcode sequences, the minimum distance of BIOUG15074-G11 to another specimen or species is 2.55% to BIOUG27197-B04. The minimum distance of BIOUG27197-B04 is 2.48% to CBG-A00991-G09. The minimum distance of CBG-A00991-G09 is 2.29% to *Aphanogmus* sp. JS-2. The distance between BIOUG15074-G11 and CBG-A00991-G09 is 3.40%.

**Condition of the specimens**.The metasomas of all specimens are separated behind the syntergum and dissected for examination of the Waterston’s evaporatorium. Head of CBG-A00991-G09 separated, one antenna and both hind legs partially missing; metasoma of BIOUG27197-B04 separated, legs partially missing or separated, one fore and one hind wing missing, mesoscutellum detached from the metanoto-propodeo-metapecto-mesopectal complex; head and metasoma of BIOUG15074-G11 separated, one fore and one hind wing missing; sixth metasomal tergite of CBG-A00991-G09 and BIOUG27197-B04 with a crack posteriorly.

## Discussion

The finding of *Aphanogmus kretschmanni* Moser, 2023 was particularly interesting due to its distinct spines on the seventh metasomal sternite. This character was unique for a ceraphronid wasp (Moser et al. 2023). Our integrative taxonomic analysis proposes at least six different species (including *A. kretschmanni*) with spiny sternite females, two of which are herein described and named. Although the species are distributed across all biogeographic realms except the Nearctic and Antarctic, they are morphologically very similar. While molecular species delimitation is clear and congruent between methods, morphological species delimitation was only possible when including both the male genitalia and the Waterston’s evaporatorium, or was even impossible with the material at hand, leading to the description of three species in open nomenclature.

**Figure 17.**
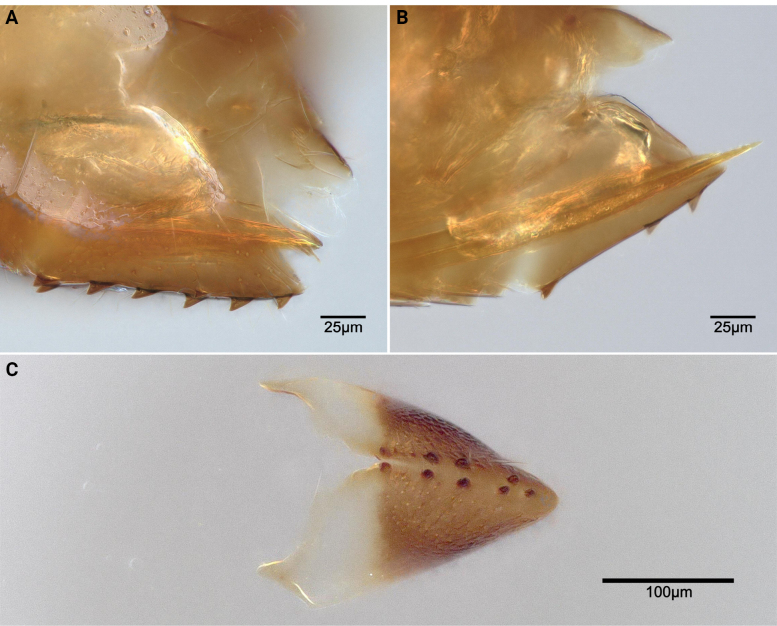
Arrangement and intraspecific variation of the spines on the seventh metasomal sternite of the female. **A, B**. Two specimens of *Aphanogmus* sp. JS-2 (**A**. Eight spines in BIOUG41413-C05, with one spine being much smaller than the others; **B**. Four spines in BIOUG41499-G12, with two spines close to each other anteriorly); **C**. Arrangement of the spines in two rows in ventral view (*Aphanogmus liceodosriosi* sp. nov. paratype CBG-A08281-A06). Images **A, B**. taken of dry specimens.

The most distinct character shared by all species treated here are the spines on the seventh metasomal sternite of the females; we here show that males do not possess spines. The spines show a great variability, also intraspecifically, in number and arrangement and cannot be used diagnostically (Fig. 17A, B). The number varied between four and eleven across all the examined specimens; the highest intraspecific variation was 5–11 in *Aphanogmus* sp. JS-3. The most frequent number of spines is six (in 11 specimens) or seven (in 8 specimens). In all specimens, two spines are situated directly next to each other anteriorly and the spines were arranged in two rows (Fig. 17C) but further arrangements varied. Regarding function of the spines, Moser et al. (2023) suggested that they either have a stabilising function during oviposition or are used for cutting into harder substrates. The association with oviposition is supported by the fact that spines are only present in female specimens. We can show this here for the first time at least for one of the examined species for which males are known (*Aphanogmus manta* sp. nov.). The function seems not to depend on the exact number of spines. However, the consistent presence of two adjacent spines arranged in two rows may support the stabilisation hypothesis, as the spines may prevent the host from rotating out of position (Moser et al. 2023). A cutting function seems more unlikely, given the sometimes low number of spines and the above described general arrangement which leads to an appearance very much different from a saw. We provide additional information on the spines regarding their morphology and their absence in males, but the function and evolutionary history of the trait remain obscure and should be subject to future research.

Herein, we use characters of the Waterston’s evaporatorium for the first time for species diagnoses, complemented by other morphological characters of body or male genitalia. The Waterston’s evaporatorium was mentioned in past species treatments (e.g., Dessart 1964, 1965; Moser et al. 2023; Salden et al. 2026b) and was suggested as a potentially species-specific character by Ulmer et al. (2021). Dessart (1992) found that the number of cells increases with size of the wasp. This conclusion cannot be supported, based on the material examined in this study. We found the Waterston’s evaporatorium to be useful to provide diagnostic characters for some of the species, but also found it harbouring some intraspecific variation and some incongruencies with the information provided by Ulmer et al. (2021). For example, the number of setae in the caudal setal row can vary greatly; for example, there is intraspecific variation of seven setae in *Aphanogmus manta* sp. nov., which differs from Ulmer et al. (2021), stating that intraspecific variation is often only one or two. Furthermore, Ulmer et al. (2021) list the distal divergence or straightening of the tergal apodemes as a typical character of the *Aphanogmus* group. This is congruent with most of the material examined here. The exceptions are *Aphanogmus liceodosriosi* sp. nov. and four additional specimens (BIOUG36037-H03, BIOUG41413-C05, CBG-A00991-G09 and BIOUG27197-B04) in which the tergal apodemes are converging. Finally, all species examined here except *Aphanogmus manta* sp. nov. have polymorphic evaporatorium cells (i.e., the size of the cells varies) with the majority of these cells being bigger than the setal sockets, which is incongruent with Ulmer et al. (2021) stating that uniformly sized cells smaller than or equal in size to the setal sockets are characteristic for *Aphanogmus*. In *Aphanogmus manta* sp. nov., the evaporatorium cells are more similar in size with less size variation and equal to the setal socket size, which is why it remains unclear if they are polymorphic or truly uniformly sized cells. In our examined material with only few males, Waterston’s evaporatoria are generally similar between males and females of the same species, although smaller and more compact in males due to the more compact tergite. A clear disadvantage of the use of the Waterston’s evaporatorium is that it requires semi-destructive dissection of the specimens, but this is also true, maybe to a lesser extent, for the male genitalia, which have proven useful for species identification (Mikó et al. 2013; Salden and Peters 2023). The evaluation of the use of the Waterston’s evaporatorium for species delimitation and diagnoses is still in an early stage. We need to gain more knowledge on the intraspecific and interspecific variation, also between females and males, to strengthen species delimitation and diagnoses based on the Waterston’s evaporatorium. Currently, countless ceraphronid species are still undescribed and females have only few diagnostic characters and can barely be matched morphologically with male specimens. Adding the Waterston’s evaporatorium as an additional taxonomic tool and integrating it with DNA sequence data, male genitalia morphology and external morphology, it may prove helpful in future studies to alleviate the taxonomic problem in the dark taxon Ceraphronidae.

We retrieved all sequences and associated specimens and metadata from BOLD and the collections of the Canadian Centre for DNA Barcoding (CCDB). The database BOLD is a comprehensive tool that makes DNA barcodes and associated metadata freely available to scientists around the world (Ratnasingham et al. 2024). By providing all data and information with open access, in combination with a first clustering of sequences into putative species via the BIN System and a preliminary taxonomic classification, BOLD is a very useful and currently underexplored resource in the process of tackling dark taxa with their vast undescribed or unrevised species diversity. In Ceraphronidae, BOLD includes 16,447 specimens/sequences from 58 countries in 2402 BINs (https://portal.boldsystems.org; accessed 02. Aug. 2025). Considering the number of known ceraphronid taxa (427), most of the putative species on BOLD will be undescribed. While assembling the dataset, we encountered occasional challenges related to specimen traceability, metadata clarity, or physical condition of the specimens. These observations highlight opportunities to further refine BOLD workflows—particularly for understudied taxa like parasitoid wasps—thereby enhancing the utility of this unparalleled repository of sequences, metadata, and vouchers for taxonomic research.

Integrative taxonomic work, exploring untapped available resources, led us to shed some more light on the diversity and morphological and evolutionary mysteries of the Ceraphronidae. The unique *A. kretschmanni* has some intriguing global relatives and there may be even more. While we have come a step closer in our species knowledge, their morphological and evolutionary mysteries are still to be solved.

## Supplementary Material

XML Treatment for Aphanogmus manta

XML Treatment for Aphanogmus liceodosriosi

XML Treatment for Aphanogmus kretschmanni

XML Treatment for Aphanogmus

XML Treatment for Aphanogmus

XML Treatment for Aphanogmus

## Additional information

### Artificial Intelligence (AI) use

The authors accept full responsibility for the content of the manuscript, including the disclosure of any use of AI.

No AI tools were used in the preparation of this manuscript.

### Author ORCIDs

J.A. Schneider https://orcid.org/0009-0000-8615-3056

T. Salden https://orcid.org/0009-0002-7993-8896

M. Moser https://orcid.org/0000-0001-7876-0278

R.S. Peters https://orcid.org/0000-0001-7784-9203

### Data availability

All of the data that support the findings of this study are available in the main text or Supplementary Information.

## Supplementary materials

Supplementary material 1 Metadata table of morphologically examined specimens Data type xlsx Explanation note The table provides the BOLD Sample ID, repository information, corresponding BIN, type status, sex, and collection data for all morphologically examined specimens. https://binary.pensoft.net/file/1728579This dataset is made available under the Open Database License (http://opendatacommons.org/licenses/odbl/1.0/). The Open Database License (ODbL) is a license agreement intended to allow users to freely share, modify, and use this Dataset while maintaining this same freedom for others, provided that the original source and author(s) are credited. Jonah A. Schneider, Tobias Salden, Marina Moser, Ralph S. Peters

Supplementary material 2 FASTA file of the alignment used for species delimitation, including additional consensus sequences for *Aphanogmus manta* sp. nov and *Aphanogmus liceodosriosi* sp. nov. Data type fa Explanation note The FASTA file contains the original alignment used for the species delimitation algorithms. In addition, a consensus sequence for *Aphanogmus manta* sp. nov. and *Aphanogmus liceodosriosi* sp. nov. is included at the end of the file. https://binary.pensoft.net/file/1728580This dataset is made available under the Open Database License (http://opendatacommons.org/licenses/odbl/1.0/). The Open Database License (ODbL) is a license agreement intended to allow users to freely share, modify, and use this Dataset while maintaining this same freedom for others, provided that the original source and author(s) are credited. Jonah A. Schneider, Tobias Salden, Marina Moser, Ralph S. Peters
